# The Mechanistic Review of the Molecular Interface of RNA-Loaded Extracellular Vesicles: Redefining Targeted Therapy for Autoimmune Disorders

**DOI:** 10.3390/ijms27104323

**Published:** 2026-05-12

**Authors:** Aliya Orassay, Naizabek Yerzhigit, Anastassiya Ganina, Elmira Chuvakova, Oleg Lookin, Abay Baigenzhin

**Affiliations:** JSC National Scientific Medical Center, 42 Abylay Khan Ave., Astana 010009, Kazakhstan; naizabek1997@gmail.com (N.Y.); anastassiya_smelova@mail.ru (A.G.); lookinoleg@gmail.com (O.L.); a.baigenzhin@nnmc.kz (A.B.)

**Keywords:** autoimmune diseases, extracellular vesicles, mesenchymal stem cells, miRNA, siRNA, RNA therapeutics, immunomodulation

## Abstract

Traditional treatments of autoimmune diseases relying on systemic immunosuppression often lack curative potential and have severe side effects. Mesenchymal stem cells (MSCs) are a promising alternative due to their immunomodulatory properties; however, whole-cell therapies have certain limitations. MSC-derived extracellular vesicles (EVs), including small vesicles—exosomes—have emerged as a safe cell-free therapeutic platform capable of crossing biological barriers and delivering bioactive cargo with low immunogenicity. Various types of RNAs abundantly produced by host MSCs represent a key element of EV content. In particular, EVs carry small RNAs, which essentially determine cellular life and fate. Our review provides a comprehensive mechanistic framework for the use of RNA-loaded EVs, specifically those carrying microRNAs (miRNAs), small interfering RNAs (siRNAs), and messenger RNAs (mRNAs), in restoring immune homeostasis. We detail the biogenesis and molecular mechanisms governing sorting of RNA into EVs, along with endogenous and exogenous engineering strategies to enhance therapeutic potency. We examine how RNA-loaded EVs modulate immunological processes like reprogramming of macrophage M1-M2 polarization, Th17/Treg balance, and suppression of inflammatory signaling pathways such as NF-κB and the NLRP3 inflammasome. We address critical translational challenges—EV heterogeneity, manufacturing scalability, and need for standardized quality control—while outlining future opportunities for RNA-loaded EV-based therapeutics.

## 1. Introduction

Autoimmune diseases are a diverse group of conditions characterized by overactivation of the immune system and cell recruitment, leading to subsequent self-tissue destruction and organ impairment [1,2]. The etiology is linked to various genetic and non-inherited environmental factors, such as epigenetic changes during cell proliferation and/or external factors such as exposure to certain drugs, heavy metals, organic compounds, and infectious agents [3]. This condition poses a significant health-care burden with an increasing global prevalence and incidence, with an annual elevation of 12.5% and 19.1%, respectively [4,5].

Current traditional first-line standard-care treatment approaches are based on non-specific, systemic modulators of the immune system via suppression of common immune function to prevent inflammation. With the knowledge that certain autoimmune diseases are linked to elevated levels of pro-inflammatory cytokines, certain biologicals and small molecule inhibitors, such as TNF-α blockers, IL-6 and IL-17 inhibitors, are being utilized to target inflammatory cytokines involved in immune dysregulation [6]. However, current strategies are not curative and are associated with several complications, including systemic side and adverse effects, a high cost, limited efficacy, and an increased risk of infection acquisition [6]. Target-directed treatment strategies are promising avenues for treating autoimmune diseases. In light of the developing understanding of detailed mechanisms of autoimmune disorders, there is a need to develop antigen-specific, immunomodulatory strategies [7]. Therefore, there is an emerging number of novel therapeutic strategies based on cellular therapy, including antigen-specific regulatory T cell (Treg) therapy [8], chimeric antigen receptor T cell therapy (CAR-T) [9], and mesenchymal stromal cells (MSCs).

Among them, recent breakthroughs in stem cell therapy have highlighted its promise as an effective treatment for autoimmune disorders. MSCs have emerged as a particularly appealing option due to their ability to control immune responses and promote tissue repair [10]. Clinical trials consistently reveal that MSC therapy alleviates disease with few side events, highlighting its safety, practicality, and therapeutic potential across a wide range of autoimmune disorders [11]. Despite these positive results, certain fundamental questions regarding optimal dosing regimens [12], the specific mechanisms of action, and long-term efficacy remain unanswered [13]. Evidence also suggests that repetitive MSC delivery may be necessary to maintain therapeutic outcomes [14].

MSCs perform broad immunomodulatory functions by regulating multiple immune cell types. For instance, they exert suppression of T cell activation and proliferation, promote Treg and Th2 cell differentiation, and switch cytokine production to an anti-inflammatory character [15,16,17]. In addition, they suppress B cell proliferation, differentiation, and immunoglobulin production, indirectly influencing them through effects on T cells and dendritic cells. Moreover, MSCs promote the development of anti-inflammatory phenotypes in macrophages and dendritic cells. Together, these actions make MSCs a promising therapy for immune-related disorders [18,19]. Despite their therapeutic promise shown in pre-clinical animal model studies, registered clinical trials had not met expectations [20]. Overall clinical outcomes regarding efficacy and the durability of treatment benefits appear to be uneven and inconsistent with the anticipated advantages [21].

MSC-based therapy presents several limitations, including host immune rejection, ectopic tissue formation arising from their differentiation potential, and adverse effects on the pulmonary microvasculature. Moreover, pro-tumorigenic activities and high variability in isolation, expansion, cellular state, and treatment potency in autoimmunity contribute to notable safety concerns that limit their clinical applicability [22,23]. With a developed understanding of the therapeutic effect of MSCs, the focus is shifted to paracrine factors secreted by MSCs [24]. Therefore, in addition to whole-cell treatment, a growing interest arises in MSC-derived extracellular vesicles (EVs), which are membrane-surrounded particles secreted (released) by cells and containing a variety of bioactive compounds such as cytokines, growth factors, and miRNAs [25,26]. Owing to their specific molecular content, EVs can modulate recipient cell functions and may serve both as therapeutic agents and pathological biomarkers [27].

The potential of EVs for drug delivery was initially identified by Valadi et al. (2007), where EVs derived from murine mast cells could transfer RNA to human mast cells, leading to the expression of exogenous murine functional proteins within recipient cells [28]. The application of EVs presents several advantages over classic MSC therapy. EVs show the capacity to exert functions similar to those of their parent cells, and show an enhanced safety profile, which is related to their biocompatibility and low immunogenicity [29]. Moreover, they possess the ability to cross biological barriers to achieve their therapeutic effect [30], offering benefits in terms of cost efficiency and ease of storage [31,32]. These vesicles play a crucial role in immunomodulatory and regenerative actions of MSCs, and are currently considered as a promising cell-free option for autoimmune disease treatment [33].

At present, EVs originating from antigen-presenting cells (APCs), including dendritic cells and MSCs, are regarded as promising cell-free strategies for the treatment of autoimmune disorders [34]. EVs derived from both immune and non-immune cells, such as stem cells, play a role in immunomodulation. Dendritic cell EVs can perform immunoregulatory activity through major histocompatibility complex (MHC) class I and class II complexes that are expressed on their surface, which consequently directly activate CD8+ and CD4+ T cells, respectively [35]. Indirect antigen presentation is achieved by the incorporation of antigenic peptide carried by the uptaken extracellular vesicle directly onto the surface of APCs via the so-called cross-dressing of immune cells [36].

An improved understanding of molecular mechanisms and signaling pathways related to autoimmune diseases has facilitated the development of EV-based therapies for restoring immune homeostasis and establishing a proper immunologic response [37]. Therefore, the immuno-regulatory function of EVs opens up new perspectives on therapeutic approaches for the treatment of autoimmune diseases [38]. Such strategies are based on loading active regulatory components into EVs to enhance their therapeutic potency [39]. For instance, dendritic cell-derived vesicles have been engineered to carry immunomodulatory proteins such as MHC-peptide complexes, co-stimulatory molecules (e.g., CD80/CD86), or pro-inflammatory cytokines that can shape T cell responses [40]. Similarly, MSC-derived vesicles have been loaded with bioactive factors, including anti-inflammatory cytokines [41], growth factors (e.g., TGF-β, HGF) [42,43], or specific miRNAs known to regulate immune cell activation and tissue repair [44]. Among those regulatory factors, RNA cargo delivery provides significant benefits in modulating functional outcomes. The administration of RNAs at low doses performs the intrinsic signal amplification from a single RNA molecule, suggesting a potential to develop RNA therapeutics with a long-lasting, robust efficacy [45]. In the context of immune modulation, they hold the capacity for programmable immune rebalancing by targeting specific genes and amplifying their regulatory effects within recipient cells, with the potential to induce antigen-specific tolerance via in situ regulatory signals or neoantigen generation [46,47].

This review provides a mechanistic insight into how RNA-loaded EVs reshape immune responses, highlighting their emerging role as cell-free therapeutic agents for restoring/repairing immune homeostasis. We discuss current endogenous and exogenous strategies for engineering EVs with diverse RNA cargos to enhance their therapeutic potency beyond their established role as biomarkers. The discussion links these mechanisms to key immunological processes central to autoimmune diseases, with particular focus on how RNA-loaded EVs modulate antigen presentation, suppress T cell activation, and reprogram inflammatory pathways such as the NLRP3 inflammasome and NF-κB signaling. In conclusion, we emphasize translational gaps and future opportunities for advancing EV-based RNA therapeutics toward real clinical deployment.

## 2. RNA-Loaded Extracellular Vesicles and Their Therapeutic Applications in Autoimmune Diseases

This section of our review provides a brief introduction to principal aspects of biogenesis of extracellular vesicles (EVs) and later shifts focus to a specific discussion on RNA-loaded EVs, including types of RNA cargo, molecular mechanisms responsible for RNA sorting and loading into EVs, and, finally, targeting strategies and administration routes for their use in autoimmune diseases. We intentionally restrained our discussion by focusing on fundamental information on EV biogenesis, as this process manages cargo loading. However, we do not delve into the size-based subtypes of EVs despite the fact that this difference may point out the EV origin (e.g., endosomal or from plasma membrane). There are several comprehensive reviews which describe the biology of EVs of different types (irrespective of their RNA content), and readers are referred to some of those sources present [48,49].

Traditionally, EVs were classified based on their sizes into three subtypes—exosomes, microvesicles, and apoptotic bodies [48]. However, recent studies are defining new EV subtypes, including stressed EVs, autophagic EVs, and matrix-bound and matrix-coated vesicles [50,51], reflecting a very important contribution of tissue microenvironment to the EVs’ secretome. Accordingly, current points of view on EV-based studies must follow the Minimal Information for Studies of Extracellular Vesicles—the guidelines aimed at standardization of terminology and proposal of strict methodological recommendations and application notes [52]. Regarding the used terminology in the present review, we use the term ‘extracellular vesicle’ whenever possible to address all subtypes (e.g., small EVs and large EVs); if we refer to a study that reported about a smaller EV subtype—exosomes—we prefer using the term ‘small EVs’ wherever possible unless the reference to the exosomal origin is critical.

EVs appear as a novel delivery system for RNA cargo transfer, providing structural stability and protection from degradation. They provide multiple advantages over other RNA delivery systems, including the ease of crossing the blood–brain barrier, low immunogenicity, intrinsic targeting, and promising immunomodulatory activity. Despite all these benefits, an essential knowledge gap persists regarding the precise function of EV-mediated RNA delivery. Natural EVs tend to carry a heterogeneous, only partially predictable cargo profile and may undergo rapid clearance, limiting their therapeutic consistency. Engineering vesicles with predefined RNA cargos provide greater specificity, enhanced stability, and precise modulation of gene expression within target cells, but still lack a precise understanding of their mechanisms. Consequently, there is a pressing need for a synthesized framework on how engineered EVs can overcome various biological barriers to deliver targeted therapeutic signals with reliability.

### 2.1. Biogenesis of Extracellular Vesicles

The mechanisms of EV biogenesis may differ depending on cell types and their signaling condition [53]. A single cell type can release multiple EV populations that differ in size, molecular composition, and surface biomarkers, underscoring the high heterogeneity of the EVs [54]. Exosomes are formed upon budding of endosomal membranes, producing intraluminal vesicles within multivesicular bodies. Multivesicular bodies can exert secretory function when fused with the plasma membrane; their intraluminal vesicles are released to the extracellular space as extracellular particles. Importantly, EV formation involves membrane budding away from the cytoplasm, a topologically distinct process mediated by the endosomal sorting complex required for transport (ESCRT).

EVs biogenesis relies on both ESCRT-independent and ESCRT-dependent pathways [55]. The ESCRT (ESCRT-0, ESCRT-I, ESCRT-II, and ESCRT-III) machinery is a central player in EV biogenesis, governing the physical processes such as membrane remodeling and scission for budding of intraluminal vesicles [56,57], as well as ensuring selective cargo loading [58,59]. The ESCRT-dependent pathway involves the PDZ domain-containing protein syntenin, syndecans, and the accessory protein Alix [60], which orchestrate specific cargo loading with ESCRT-III-driven membrane remodeling [56]. Additionally, ESCRT-I component Tsg101 and ESCRT-II component Vps22 were identified to be important in syndecan–syntenin exosomes formation [60,61].

Furthermore, tetraspanin-enriched microdomains and ceramide-mediated membrane curvature are examples of ESCRT-independent processes that can function alone or in coordination with ESCRT pathways. ESCRT-independent mechanisms include lipid components such as ceramide, involved in selective cargo sorting. A cone-shaped structure of ceramides causes negative membrane curvature and favors inward budding of the endosomal membrane [62]. Tetraspanins such as CD9, CD63, and CD81 are highly present in exosomes and are utilized as biomarkers for exosomes [63,64]. Beyond their role as biomarkers, they also function as platforms for protein clustering [65], promoting membrane curvature [66], which facilitates vesicle release [63,67]. Furthermore, tetraspanins regulate the sorting of cargos into EVs via their interaction with integrins and immunoreceptors, which contributes to functional heterogeneity among vesicle populations [68,69]. In addition to their role in sorting, tetraspanins are involved in EV targeting and uptake by recipient cells, further shaping EV-mediated intercellular communication [70].

Secretion of EVs is also dependent on the cytoskeleton. For example, microtubules carry the multivesicular body to the plasma membrane. The actin filaments also need to be rearranged through the action of Arp2/3, with cortactin stabilizing branched actin to enable docking of multivesicular bodies [71]. Other proteins involved in EV release include small GTPases of the Rab family, such as Rab27A/B [72,73], Rab7 [74], Rab11 [75], and Rab35 [73,76]. In addition, different N-ethylmaleimide-sensitive factor attachment protein receptor (SNARE) family members, including vesicle-associated membrane proteins 2/3/4/7/8 (VAMPs), have been implicated in the fusion of multivesicular bodies with the plasma membrane [77]. Moreover, the EV secretion can be controlled by ubiquitin-like protein Interferon-Stimulated Gene 15, which promotes degradation of Tsg101, further hindering formation of vesicles [78]. Collectively, these mechanisms work together to tightly regulate EV biogenesis and cargo composition, which enables EVs to carry out a range of biological functions. The defects in this complex lead to abnormal EV secretion and pathology progression.

### 2.2. Types of RNA Cargo in Extracellular Vesicles

RNA-enriched extracellular vesicles carry diverse classes of RNA cargo such as messenger RNAs (mRNAs), microRNAs (miRNAs), small interfering RNAs (siRNAs), and long non-coding RNAs (lncRNAs), which are collectively involved in regulating gene expression, cellular processes, and immune signaling in recipient cells [79]. Transferring of those RNA cargoes through EVs enables regulation of inflammatory pathways, immune cell differentiation, and cytokine production, highlighting their therapeutic potential in autoimmune diseases aside from their established use as biomarkers [80,81,82].

Some RNAs in extracellular vesicles, such as mRNA and miRNA, are considered functional when transferred to recipient cells, while others, such as PIWI-interacting RNAs and vault RNAs, fail to perform intercellular interaction. Some fragments of RNAs, including tRNA, mRNA, and rRNA fragments, demonstrate functionality, while non-functional fragments degrade. Notably, miRNAs are particularly significant immune homeostasis regulators among EV-associated RNA cargos. These small non-coding RNA molecules (21–23 nucleotides), acting as post-transcriptional regulators of gene expression, serve as essential players for the onset and course of several autoimmune conditions [83]. These miRNAs play a critical role in regulating immune responses and the development of immune cells. Disruption of the miRNAs’ balance can lead to autoimmune diseases, making miRNAs a potential target for therapy [84]. For instance, in the Sanroque mouse model, researchers revealed a miRNA-mediated pathway in which miR-101 is essential for Roquin-mediated degradation of mRNA for the Inducible Costimulator Protein (ICOS). This process prevents the build-up of lymphocytes and the development of lupus-like autoimmune diseases. This finding highlights the importance of miRNA-mediated post-transcriptional regulation in maintaining immune tolerance [85].

It has been found that miRNAs play a vital role in the proper functioning of the regulatory T cells. The evidence comes from studies showing that the selective knock-out of miRNA processing enzyme Dicer in the regulatory T cells impairs their peripheral stability; the suppressive capacity of the immune response, the balance of the expression of the forkhead box protein P3 (FoxP3), and the onset of severe systemic autoimmune disease can all be affected [86,87].

In this realm, EVs of MSCs carrying miRNAs have emerged as promising cell-free therapeutic candidates, which have shown positive effects in a variety of biological systems and diseases [88,89]. The miRNA contained in these EVs has been shown to regulate the immune response, which in turn modulates autoimmune reactions through the fine-tuning of inflammatory responses. Some of these miRNAs are known to augment inflammatory responses by down-regulating anti-inflammatory targets, while others protect against inflammatory responses by targeting key pro-inflammatory signaling pathways [90]. Delivering miRNAs through exosomes offers multiple benefits compared to the direct injection (e.g., systemic or intra-tissue) of the miRNA-containing content. Notably, exosomes facilitate direct cellular uptake of the miRNAs and limited enzymatic degradation (due to the lipid membrane), both of which elevate their bioavailability [91,92]. In addition, exosomal miRNAs have shown the potential to modulate immune responses and affect the development of autoimmune disease, as the miRNAs can either exacerbate the disease by reducing the expression of anti-inflammatory genes or prevent the disease by targeting the pro-inflammatory signaling pathways.

Small interfering RNAs (siRNAs) regulate gene expression by targeting mRNA cleavage and degradation mediated by catalytic RISC protein, which consequently prevents mRNA translation, and silences specific genes by performing chromatin modification [93,94]. Based on this mechanism, siRNA-based therapeutics are being showcased as promising strategies for various treatments, including autoimmune diseases [95,96]. While siRNA therapies demonstrate significant promise, the practical application is limited by their rapid degradation in the body, off-target effects on other cells, and low uptake capacity by their target cells [97]. Despite this shortcoming, siRNA therapeutics hold the key to the treatment of autoimmune diseases by specific targeted silencing of key genes involved in the inflammatory cascade, such as TNF-α and NF-κB. This action leads to a reduction in pro-inflammatory cytokine production, modulation of immune cell activity, and final prevention of tissue damage, as shown in rat model of rheumatoid arthritis [98]. When delivered via nanoparticles or other carriers, siRNAs achieve precise regulation of pathogenic immune responses while minimizing off-target effects, making them particularly attractive for autoimmune disease treatment [99]. Both viral and non-viral carriers have been investigated to facilitate siRNA-targeted delivery for different therapeutic applications [100,101]. EVs of various sizes, including small EVs, have recently gained attention as natural delivery vehicles that can transport functional siRNAs into recipient cells, enabling effective gene silencing [102].

Despite the progress of EV-siRNA as a therapeutic agent, its clinical application is currently limited [103,104]. However, in preclinical autoimmune disease models, siRNA delivery has demonstrated significant therapeutic potential. For instance, siRNA of Endoplasmic Reticulum To Nucleus Signaling 1 (ERN1) delivery by cationic polymer-based nanoparticles exhibited efficacy in autoimmune disease treatment by targeting type 1/3 inositol 1,4,5-trisphosphate receptor (IP3R1/3) and regulating calcium ion concentration in the mouse models of collagen-induced arthritis and inflammatory bowel disease. As a result, it promotes anti-inflammatory M2 macrophage polarization and inhibits MyD88-dependent toll-like receptor (TLR) signaling [105]. Preclinical studies have also demonstrated that EV-encapsulated siRNAs can functionally silence pro-inflammatory cues such as TNF-α in both immune and tissue cells. This reduces harmful immune reactions in autoimmune and inflammatory disease models [106]. A notable study in rheumatoid arthritis models took this concept further by integrating a cryomicroneedle-mediated transdermal delivery system to enhance tissue penetration, stability, and TNF-α targeting efficacy of siRNAs extracted from milk [107].

The successful development of an mRNA vaccine for the SARS-CoV-2 virus (COVID-19) demonstrated the potential of mRNA-based vaccines and therapies to improve health outcomes [108]. The ability of mRNA to encode for various proteins involved in immunomodulation has widened its application in the treatment of cancer and other diseases [109]. While mRNA-loaded EVs have been gaining significant attention in the treatment of cancer and viral infections, there is a limited number of studies investigating their therapeutic effects on autoimmune diseases [110]. The mRNA-lipid nanoparticles (LNPs) have emerged as an innovative and promising therapeutic strategy for autoimmune diseases. For instance, Liu et al. used lipid-polymer nanoparticles to deliver mRNA encoding FcγRIIB, which is presented as an inhibitory receptor in the B cell surface and the loss of which is related to autoimmune disease development, to relieve symptoms in the mouse model of collagen-induced arthritis [111]. Early studies revealed that EVs mediate mRNA transfer between cells, where the mRNA is translated into functional protein upon entering the recipient cell [112]. Interestingly, Tsai et al. (2021) highlight elevated expression of mRNA by mRNA-loaded small EVs compared to mRNA-loaded LNPs without eliciting adverse effects, suggesting that EVs may serve as an efficient therapeutic platform for mRNA delivery [113]. However, variability in the mRNA profile within EVs raises concern about therapeutic consistency, requiring further characterization of targeted mechanisms. Furthermore, mRNAs being much longer than small RNAs presents difficulties for their natural non-facilitated uptake by EVs [114]. On the other hand, chemical modifications of EVs to enhance mRNA loading may have adverse effects (we address this below).

Long non-coding RNAs (lncRNAs) are RNA transcripts with a length of more than 200 nucleotides [115]. They have been suggested as biomarkers of many autoimmune diseases, including rheumatoid arthritis, systemic lupus erythematosus, Sjögren’s syndrome, and others, due to their involvement in the differentiation and activation of immune cells [116,117,118]. They show better enrichment in EVs compared to the cells of origin, suggesting their active natural loading capacity into the EVs [119]. lncRNA can interact with DNA and other RNAs, such as mRNA, miRNA, and circRNA. Upon binding to mRNA, it affects the translation or acts as a sponge to block the miRNA binding site, suppressing miRNA function [120]. Taking into account that lncRNAs are involved in many physiological processes, the delivery of lncRNAs via EVs is shown as an effective treatment strategy for various pathological conditions. For example, Tao et al. (2018) have demonstrated that lncRNA H19 delivered by extracellular vesicle mimetic promotes angiogenesis through regulating the insulin-PI3K-Akt pathway [121]. Other researchers revealed that RNA of metastasis-associated lung adenocarcinoma transcript 1 (MALAT1) in small EVs can lead to endothelium formation through suppression of miR-92a expression to prevent miR-92a from suppressing KLF2 [122]. Besides the therapeutic role of lncRNAs in tissue repair and regeneration, they show therapeutic effects in several cancer types through molecular mechanisms, including miRNA sequestration, direct mRNA or protein interaction, and inhibition of pro-survival signaling pathways in tumor development [123,124,125]. Exosomal lncRNAs function as “biological repair kits” for the treatment of autoimmune diseases by using their capacity to selectively “sponge” miRNAs and stimulate angiogenesis via pathways such as PI3K-Akt. This method does not simply silence the pro-inflammation signals that lead to self-harm but also activates the regenerative machinery, which assists in restoring the damaged tissues caused by chronic inflammation.

Circular RNAs (circRNAs) are covalently closed, highly stable RNA molecules generated by back-splicing. In addition, circRNAs are highly likely to be incorporated into EVs, making them good candidates for biomarkers [126]. They function in the regulation of gene expression through binding to RNA-binding proteins and acting as miRNA sponges, thereby influencing immune-related signaling pathways and inflammatory responses characteristic of autoimmune diseases [127]. Transfer RNAs (tRNAs) have also been identified to be present in abundance within EVs compared to other cellular RNAs [128,129]. Besides full-length tRNA, tRNA fragments are also represented in EVs, which may function to inhibit protein translation [130,131]. Other RNAs involved in replication and post-transcriptional modification have been identified in EVs, including small nuclear RNAs (snRNAs), small nucleolar RNAs (snoRNAs), Y RNAs, and small Cajal body-specific RNAs (scaRNAs), etc. [132].

### 2.3. RNA Loading into Extracellular Vesicles

Understanding of the natural processes of EV biogenesis assists the development of strategies for engineering RNA cargo-containing EVs for disease treatment, such as autoimmune diseases. The cargo selection process is well characterized for membrane proteins, where proteins are ubiquitylated and recognized by the ESCRT complex and internalized. However, cytoplasmic RNAs cannot be directly recognized by the complexes, as it is shown in the classical biogenesis pathway, implying the use of additional molecular adaptors for RNA incorporation.

#### 2.3.1. Molecular Mechanisms of RNA Sorting and Loading into Extracellular Vesicles

Loading of RNA into EVs can be achieved by passive loading, where loading is driven by intracellular RNA abundance. Accumulating evidence indicates that in addition to passive loading based on RNA abundance, active strategies can also be applied to enhance loading efficiency. Active loading is mediated by selective recognition of RNA cargo through regulatory RNA-binding proteins, including Argonaute-2 (AGO2) [133], annexin A2 [134], major vault protein (MVP) [135], heterogeneous nuclear ribonucleoproteins A2/B1 (hnRNPA2B1) [136,137], activity-regulated cytoskeleton-associated protein 1 (Arc1) [138], Y box binding protein 1 (YBX1) [139], synaptotagmin-binding, cytoplasmic RNA-interacting protein (SYNCRIP) [140], and the La protein [141], each recognizing and binding to RNA sequence motifs and/or secondary structures. Specific motifs, such as EXOmotifs and CELLmotifs, are recognized by those RNA-binding proteins and ensure selective RNA loading into small EVs [142]. Computational analyses have identified specific conserved sequence motifs enriched in EV-associated RNAs, suggesting the presence of cis-acting signals that direct RNA sorting into vesicles. These motifs often co-occur within the same transcript and are particularly enriched in lncRNAs, supporting their potential as selective exosomal RNA packaging [143].

It has been shown that RNA packaging is not restricted to sequence motifs, but that many modifications in RNAs and RNA-binding proteins play a role. For instance, SUMOylation can influence the loading of miRNAs through the hnRNPA2B1 protein, and phosphorylation can influence RNA packaging, such as that of exosomal 5′pppRNA in the context of latent infection by Epstein–Barr virus [144]. Conjugation of RNA-binding proteins, heterogeneous nuclear ribonucleoprotein K (hnRNPK) or scaffold-attachment factor B (SAFB) to a lipidated form of microtubule-associated protein 1A/1B-light chain 3 (LC3 protein)—a member of the ubiquitin-like modifiers superfamily—also contributes to effective loading of miRNAs into EVs [145]. The association of miRNAs with these RNA-binding proteins utilizes autophagy machinery for their selective and non-random sorting into EVs for further secretion, representing promising therapeutic strategy [146]. Of note, RNA-binding proteins contribute to RNA loading both directly, through delivering RNAs to the sites of EVs biogenesis, and indirectly through the regulation of RNA content in various cellular compartments [147].

In addition, engineered EVs were developed to selectively encapsulate therapeutic mRNAs using a DNA aptamer that inhibits translation and promotes EV sorting [148,149]. For example, delivery of IL-10 mRNA via CD9-ZF-engineered EVs significantly increased IL-10 levels, suppressed intestinal inflammation, and ameliorated dextran sulfate sodium (DSS)-induced colitis in mice [150]. The development of EVs coated by MS2 proteins, which provides strong binding to the MS2-RNA aptamer, has allowed a significant enhancement of active loading capacity of the vesicles [151]. Covalent conjugation of highly myelin-affine LJM-3064 aptamer to the exosomal surface has been successfully tested for targeted delivery of the aptamer to induce remyelination in vivo in mice, and is proposed as a therapeutic platform in treating multiple sclerosis [152,153].

In general, there are two main ways to load RNA cargo into EVs. In endogenous loading, the delivery of cargo is mediated through transfection and co-incubation. The EVs from the transfected cells are then collected for further applications. Exogenous loading is the collection of EVs from parental cells, later inducing RNA entry by electroporation, ultrasound, or co-incubation. We discuss these two main loading strategies in more detail in the following subsections.

#### 2.3.2. Endogenous Loading of RNA Cargo into Extracellular Vesicles

Endogenous loading is achieved by genetically modifying the parent cell to overproduce RNA content; the process relies on the natural sorting machinery for cargo loading. This approach is usually preferred over exogenous loading, since RNA cargo is protected by vesicle membrane during biogenesis, compared to exogenous methods such as electroporation that may cause membrane damage of produced vesicles. On the other hand, transfection of the parent cells is often implemented to produce RNAs with specific features in abundance. Such an engineering approach inherently poses safety concerns and is accompanied by EV contamination by plasmid content, etc. [154,155]. In addition, endogenous loading represents a better and more efficient approach to generate off-the-shelf products for further EV-based therapies [156].

In studies dedicated to exosomal RNA delivery for the treatment of autoimmune diseases, there is widespread application of endogenous cargo loading techniques. For instance, the study by Chen et al. (2018) utilizes a genetic modification approach for exosomal engineering [157]. Instead of loading the cargo into already-formed EVs, researchers engineered the donor cells (MSCs) to overexpress the desired miRNA. This allows the cells’ internal machinery to handle the sorting and packaging of the cargo into the vesicles [157]. Similarly, Li et al. (2021) transfected bone marrow mesenchymal stem cells with miR-21 mimic to overexpress miRNA, which is naturally packaged into secreted EVs [158]. Nucleic acids can be encapsulated in small EVs through the transfection of cells that release the small EVs with plasmids containing the necessary encoding RNAs [159]. For example, Meng and Qiu (2020) have transfected MSCs with miR-320a mimics using Lipofectamine 2000 [160]. In an attempt to resolve the long-standing issue of loading efficiency of large nucleic acids, Zhang et al. (2021) developed a method of selective encapsulation using a DNA aptamer bridge [150]. In this method, the authors engineered the donor cells to express a chimeric protein comprising a CD9 zinc finger domain, which was able to encapsulate mRNAs, like IL-10 [150]. Beyond protein–protein bridges, active recruitment can be achieved by hijacking the biochemical properties of the multivesicular body membrane. The RNA affinity to lipid-raft regions can be increased through RNA-binding proteins and specific sequence motifs in RNA [161]. This chemical association facilitates vesicular uptake in essentially the same way as it has been demonstrated for various transmembrane proteins [48].

The purpose of engineering EVs with selectively overexpressed RNA cargo is to further deliver the therapeutic content to target cells. Notably, passive endogenous loading uses the constructs, where overexpressing RNA vectors passively increases RNA loading, whereas active loading is characterized by producing recombinant fusion through receptor-binding domains [162].

#### 2.3.3. Exogenous Loading of RNA Cargo into Extracellular Vesicles

EVs can be loaded exogenously, following isolation from target cells, through passive incubation with cargo or by active physical loading methods such as electroporation, sonication, surfactant treatment, or freeze–thaw cycling [163,164]. For exogenous incubation of RNA cargoes to EVs, the EVs should first be isolated from parent tissues and body fluids by using proper isolation methods such as ultracentrifugation, size exclusion chromatography, etc.

While passive incubation is widely used for small-molecule drug loading (e.g., paclitaxel and doxorubicin in cancer treatment) [165,166], loading RNAs into isolated small EVs often requires more sophisticated methods. Xie et al. (2025) demonstrated the successful loading of miRNA let-7f-5p into labial gland MSC-derived EVs using a direct incorporation method via Exo-Fect reagent for their further use to treat Sjögren’s syndrome [167]. Researchers have previously shown that Exo-Fect was the most efficient method among others, giving more than 50% loading efficiency into exosomes and diminishing the enzymatic breakdown of the exosomes during intracellular transport [168]. Similarly, Zhang et al. (2024) employed active chemical mediators to load siRNAs for targeted gene silencing in hepatic macrophages, underscoring the potential for tailored RNA-interference therapies in autoimmune diseases [169]. Furthermore, Mi et al. (2024) employed a specialized exosomal RNA loading kit to exogenously encapsulate miR-451a into pre-isolated human umbilical cord MSC-derived exosomes [170]. Using a chemical ‘Exo transporter’ and a two-hour incubation period, they achieved efficient cargo loading that proved more effective than naturally occurring exosomes for alleviating synovial pathology in rheumatoid arthritis models.

As passive incubation is generally less efficient for highly charged nucleic acids compared to small-molecule drugs, specific chemical modification of the RNAs may be of assistance. Han et al. (2024) demonstrated that modifying siRNA with a hydrophobic cholesterol moiety (siTNF-chol) allows for efficient exogenous loading into milk-derived EVs through simple passive incubation, effectively anchoring the cargo into the vesicle membrane for oral delivery [106]. Similar to this strategy, conjugation of structurally modified siRNAs with cholesterol has been shown to elevate their uptake by exosomes both in vitro and in vivo [171,172]. Importantly, the incubation ratio of siRNA to exosome is not linearly related to the final siRNA concentration in the exosomes. Instead, there is a saturation limit indicating that further increase in siRNA amount in the loading medium does not produce higher accumulation in the exosomes [171]. This finding reflects the inherently limited capacity of small and large EVs to accumulate a substance of interest, like RNA, which should be taken into account when EV-based therapy is translated into clinical practice.

Other researchers have employed active physical methods such as sonication to encapsulate TNF-α siRNA into milk-derived exosomes with a loading efficiency of approximately 21%. To overcome the challenges of systemic delivery, these siRNA-loaded milk-derived exosomes were integrated into a cryomicroneedle patch for transdermal administration [107]. The results are consistent with other studies, where sonication yields a higher loading capacity compared to other simple incubation strategies [173,174]. Interestingly, in most of the studies that utilize siRNA-loaded EVs for the treatment of autoimmune diseases, the loading is handled by using exogenous loading techniques such as passive co-incubation with RNA or sonication. This is not surprising given that siRNAs are easy to load via the above-mentioned techniques due to their small size (20–25 nucleotides) [175,176].

#### 2.3.4. RNA Enrichment Strategies

The targeting modifications mostly rely on modification of the EV structure and membrane composition to improve homing to and effective interaction with target cells. However, the cargo itself may be less effective when present at small concentrations within EVs. The term so-called “fold enrichment” refers either to the improvement of EV loading by specific substances or to the increased concentration of specific EVs in the final product. In both cases, the higher concentration of active agents results in elevated beneficial outcomes of EV-based therapeutic intervention. Compared to other molecular types like large proteins, small RNAs (miRNAs, siRNAs, etc.), and long RNAs (lncRNA, circRNAs, etc.) are able to accumulate in the EVs in relatively large amounts [177]. The distribution of RNA subtypes, such as miRNAs and lncRNAs, varies among EV populations and is influenced by vesicle size, with miRNAs being more abundant in small EVs [178]. Interestingly, some human MSC-derived miRNAs may be overrepresented in EVs compared to parent cells, whereas others are underrepresented. However, the majority of miRNAs appear to be expressed on similar levels between all-sized MSC-EVs and MSCs [179]. This may imply different active loading mechanisms for: (a) small and long RNAs—likely simply due to their different molecular weight—and (b) certain miRNAs—probably via different affinity to the EV membrane components. However, compared to the parent MSCs, most of the studied miRNA content in the MSC-derived small EVs is depleted [180]. In addition, the proportion of EVs that are enriched with miRNAs can be as low as 10% of all EVs; it can be considered as a distinct subpopulation of EVs with specific functionality and regulatory potential by means of utilization of intracellular transcriptional/translational machinery [181].

The enrichment of miRNA content is of ultimate importance to overcome the problem associated with the above-mentioned miRNAs underrepresentation in EVs, especially shown in naturally produced vesicles. As a complementary for endo- and exogenous loading which may, for example, utilize a specific cell line transfected by a viral vector and overexpressing miRNAs of interest in a large-scale quantity [182,183], novel techniques have been developed to produce highly RNA-enriched EVs. This includes the use of magnetic particles that selectively bind to the lipid bilayer of EVs, which can subsequently be used to saturate the EV content [184]. The magnetic bead-bound affinity-based saturation of miRNA enables a sufficient amount of EVs to be ontained in a plasma sample as small as 100 uL. Finally, this strategy inherently has higher specificity compared to a traditional ultracentrifugation method, eliminating a chance of contamination by similar density compounds like lipoproteins [184]. Other methods have been proposed such as calcium chloride-assisted transfection-like loading, which represents direct loading of miRNAs into isolated small EVs instead of transfecting native cells [185].

On the other hand, some types of RNAs may naturally accumulate in the EVs in abundant concentrations, resulting in a very high fold enrichment. For example, lncRNAs related to virus-like protein families demonstrate huge ability for fold enrichment in small EVs [186]. In mice, it has been found that lncRNA of retroviral origin, VL30, is enriched to over 200-fold in the EVs derived from dendritic cells compared to the content in parental cells [187]. Moreover, sequence analysis of EV-encapsulated RNA for VL30 revealed that it contains a repetitive motif exhibiting immunomodulatory properties, implicating its involvement in autoimmune responses. Of interest, this ability may be beneficial in designing strategies for targeted RNA delivery that is based on utilizing a ‘humanized’ capsid particle that specifically targets a certain type of cells [188]. Similarly, small EVs from brain tissue were shown to be highly enriched by RNAs that encode human-specific long interspersed nuclear element 1 (LINE-1) retrotransposons [189], which are known to be the only active regulators of the ‘copy-and-paste’ mechanism of epigenetic modification due to their self-replication and ability to affect expression of proteins involved in pathogenic processes [190]. Notably, this specific EV content may be of great importance in the engineering of therapeutic EVs.

We summarized available data for RNA cargo loading and purification of EVs obtained from different sources (including MSCs and immune cells) and loaded by miRNAs or other types of RNAs in Table 1. Note that in previous years, the most frequent method to load the EVs by a target (often modified) miRNA was a non-viral transfection (generally, Lipofectamine 2000 is used). This simplifies the overall procedure while providing a robust approach to get required amounts of RNA-loaded EVs. Natural enrichment of EVs is not commonly employed due to the requirement for prolonged cultivation and its strong dependence on environmental condition and secretory activity of the parent cells. However, endogenous production of RNA-loaded EVs by the living cells still remains a preferred method.

### 2.4. Targeting Ability of Extracellular Vesicles in Autoimmune Diseases

#### 2.4.1. Cell Origin and Homing Tendency of Extracellular Vesicles

Studies demonstrate that cells of various origins exhibit distinct homing behaviors within an organism. Different cell types, including stem cells, progenitor cells, and immune cells, have a natural homing capacity to their respective niche [211]. For example, this can be evident if dendritic cells are administered into the bloodstream and then tend to accumulate in immune organs such as the spleen [212]. Similarly, the homing of different types of EVs has also shown a natural biodistribution pattern depending upon the type of originating cells, even when delivered through the same administration route [213]. For example, immune cells in autoimmune diseases like Sjögren’s syndrome move directly to tissues. This movement reflects how the disease develops and demonstrates how disease areas affect cell movement, a factor important for EV-based targeting [214]. However, several studies report that EVs demonstrate limited autonomous homing capacity, suggesting that active targeting strategies remain necessary [215]. While intravenous injection remains the most clinically relevant administration route, integrating this natural homing capacity with rational delivery design may enhance targeting efficiency. For example, gingival MSCs (GMSCs) come from neural crest cells and are constantly found in a permanently inflamed setting. They make EVs that carry regulatory substances, like miR-148a, which controls the Treg/Th17 balance using the IKKβ/NF-κB pathway, and have also shown better therapeutic effects in rheumatoid arthritis models [204].

Emerging evidence further indicates that EV source selection in preclinical studies often follows disease- and organ-specific usage patterns rather than a uniform sourcing strategy. In many cases, investigators preferentially select EVs from a tissue that is biologically or developmentally related to the affected organ, reflecting an assumption of tissue-matched functional specialization or enhanced therapeutic relevance [216].

Most studies using RNA-loaded EVs in autoimmune disease models employ MSCs as the primary EV source, owing to their proliferative capacity, low immunogenicity, and well-characterized safety profile [217]. Local inflammation can modify MSC phenotype and EV cargo, and several studies report that MSC-EVs preferentially accumulate to injured or inflamed tissues, suggesting a degree of inflammation-associated tropism that may be leveraged for targeted delivery in autoimmune disorders [218]. Beyond biodistribution, the choice of the parent cell is also critical for determining the immune-modulatory function of EVs. Research studies demonstrate that the immunoregulatory properties of MSCs depend on their tissue of origin and environmental conditioning [219].

Despite the promising targeting and immunomodulatory potential of MSC-derived EVs, heterogeneity in cell origin and batch variability creates challenges in quality control, management, and scalability. Umbilical cord MSCs (UCMSCs) exhibit higher proliferative capacity compared to other MSCs derived from adult tissues, which may favor their use in scalable therapeutic manufacturing [220,221]. UCMSCs-EVs also demonstrate an increased anti-inflammatory effect for autoimmunity treatment, comparable to other EV sources with immunomodulatory ability [222]. Inflammation suppression can also be attributed to upregulated miRNA content upon inflammatory priming conditions [223]. In addition, EVs from UCMSCs were shown to promote wound healing and angiogenesis to mitigate fibrosis, the effect attributed to embedded RNA content [224,225].

Many such studies in autoimmune disease models continue to employ bone marrow mesenchymal stem cells (BMSCs)-derived EVs, owing to their extensive characterization, consistent quality profile, and suitability for autologous application without eliciting donor-specific immune reactions [226]. Compared to UCMSCs, the secretome of BMSCs exhibits a stable and stronger anti-inflammatory profile after hypoxic priming [227]. Compared to EVs derived from adipose-derived mesenchymal stem cells (ADSCs), BMSC-EVs regulate the immune system more effectively, particularly in early passages and under an inflammatory environment. In such cases, BMSC-EVs raise macrophagal M2 gene expression (IL-10, Arg-1, TGF β) with passage-dependent loss of potency [228]. BMSC-EVs and ADSC-EVs performed angiogenesis via inducing secretion of cytokines and factors promoting vascular cell formation [229,230]. BMSC-EVs also displayed prominent osteogenesis capacity [231].

Finally, EVs from emerging sources may provide source-specific advantages. For example, EVs derived from dental pulp stem cells (DPSCs) showed stronger immunomodulatory effects than those from BMSCs, including greater promotion of Treg polarization, Th17 inhibition, and shifts toward anti-inflammatory cytokine profiles. The DPSC-derived EVs also induced more CD4^+^ T cell apoptosis and comparably suppressed proliferation, suggesting a superior therapeutic prospect for immune-related diseases [232].

#### 2.4.2. Route of Administration and Biodistribution of Extracellular Vesicles

Consistent with prior work, the studies emphasize that systemically delivered EVs are rapidly cleared by the mononuclear phagocyte system, such as Kupffer cells in the liver or splenic macrophages, thereby greatly restricting the proportion of vesicles that reach peripheral disease-relevant tissues [233]. This hepatic sequestration represents a major barrier for EV-based RNA delivery outside liver-targeted indications. Although surface engineering approaches can partially reduce nonspecific uptake, the study highlights that EV biodistribution is still strongly governed by the combination of cell source and route of administration, rather than engineering alone [233,234]. This indicates that administration routes may be strategically selected to minimize off-target accumulation and enhance delivery to disease-relevant tissues.

Across autoimmune disease models, EV-based therapies have been delivered via multiple administration routes with demonstrated immunomodulatory effects. Local or transplantation-associated delivery of BMSC- and peripheral blood mononuclear cell (PBMC)-derived EVs suppressed immune activation by inhibiting PBMC proliferation and enhancing regulatory T cell function following islet transplantation [235]. Systemic intravenous administration of IFNγ-stimulated MSC-derived EVs produced sustained clinical improvement and reduced neuroinflammation and demyelination in experimental autoimmune encephalomyelitis [236].

Oral delivery of bovine milk-derived EVs delayed the onset of rheumatoid arthritis and reduced inflammatory pathology and cytokine levels [237], while intraperitoneal injection of ADSC-derived EVs exaggerated regulative T cell populations in autoimmune type 1 diabetes without affecting lymphocyte proliferation [238]. Differences in biodistribution are observed within EVs derived from different sources with different routes of administration. This underscores the importance of optimizing delivery strategies and the selection of appropriate EV sources to ensure tissue-targeted effective delivery.

Notably, while intravenously administered EVs were shown to aggregate further within the liver and spleen organs, intraperitoneal and hypodermic EV administration demonstrates less accumulation in these organs. Instead, significant accumulation in the gastrointestinal tract and pancreas is evident [233]. With the advancement of EV-based research, scholars are drawing attention to more cost-effective and accessible solutions, such as the use of plant and milk products as natural EV sources. Moreover, in order to address targeting issues, the surface modification of EVs by using ligands, pH-responsive motifs, and magnetic materials is widely practiced to enhance EV targeting to specific cells.

#### 2.4.3. Strategies to Improve Targeting of Extracellular Vesicles

It is well known that EVs, particularly engineered EVs, possess low immunogenicity, intrinsic biocompatibility, and the partial targeting capability conferred by surface proteins. EV-based delivery of RNA therapeutics to specific tissues, therefore, represents an attractive strategy for advancing genetic therapies in autoimmune diseases. However, the use of unmodified or naïve EVs is often insufficient to achieve meaningful target enrichment, as EVs are prone to nonspecific uptake and rapid sequestration by off-target tissues [239]. This limitation underscores the need for rational targeting strategies that improve tissue specificity and therapeutic delivery efficiency.

Targeting performance can be enhanced by modifying surface components on EVs, which can be achieved through transfection of cells to allow protein attachments to the EV’s surface, or by direct modification by chemical and physical means. A range of targeting engineering strategies has been developed for therapeutic RNA delivery, including chemical covalent modifications [104], non-covalent modifications [240], enzymatic surface functionalization [241,242], the use of exosome hybrid systems [243], and co-incubation [244] and PEGylation [245] approaches, many of which have been successfully applied in cancer treatment.

By contrast, targeted delivery of RNA-loaded EVs in autoimmune diseases remains comparatively underdeveloped, representing a key opportunity for innovation. Recent advances provide early proof-of-concept: Niu et al. (2025) demonstrated improvement in ameliorating systemic lupus erythematosus (SLE) by engineered Ago-Exos with preferential targeting of immune cells [246]. Targeting was achieved through aggregation-culture-induced biological reprogramming of exosomes/EVs, attributed to enhanced expression of different molecules such as intercellular adhesion molecule 1 (ICAM-1) essential for immune cell targeting. In multiple sclerosis, EDC/NHS chemistry was utilized for chemically conjugating carboxylic acid-functionalized DNA aptamer (LJM-3064) with amine groups on exosomal surface to promote myelin binding, enhancing targeting specificity [247].

One of the strategies is surface engineering of EVs by chemical means for targeting specificity. In rheumatoid arthritis, the FPC-Exo/Dex active targeting drug delivery system was created by modifying the surface of EVs with a folic acid (FA)-polyethylene glycol (PEG)-cholesterol, which enables enhanced accumulation and reduction of joint inflammation [248]. Other researchers developed neutrophil-derived EVs functionalized with sub-5 nm ultrasmall Prussian blue nanoparticles (uPB-Exo) by using click chemistry. These engineered EVs selectively targeted inflamed joints via neutrophil-targeted biological molecules inherited from neutrophils, alleviating rheumatoid arthritis [249]. Additional studies indicate that polyarginine peptide incorporation into the surface of M2 macrophage-derived EVs demonstrates an increased targeting effect and cellular uptake rate, showing accumulation at the site of inflammation [250].

#### 2.4.4. Potential Limitations of Engineered Cargo Loading to Extracellular Vesicles

The loading of RNA content into EVs via classic electroporation or chemical methods remains a challenging strategy. Direct loading techniques, such as electroporation and reagent-assisted loading strategies, are technically complex and require multiple rounds of separation and purification. In addition to increasing procedural complexity, the repeated processing and reliance on high-speed centrifugation may negatively impact vesicle integrity and functional quality and encourage their loss [251]. For instance, the use of electroporation of EVs with siRNA has been proven to form aggregates and precipitates on the EV surface, reducing loading capacity and quality of the final product [252].

On the other hand, chemical modifications of the EV membrane targeted to facilitate RNAs loading may represent potential pitfalls due to possible compromising of the structural integrity of the membrane and toxicity issues. The lipid content of EVs membrane affects its physical properties (fluidity) that directly contribute to the RNA–lipid interaction and therefore the binding of RNA oligomers to the lipids; in addition, this may affect the activity of loaded RNAs [253]. Additionally, hydrophobic modification of the EVs membrane may promote fusion of lipophilic content occurring spontaneously, thus further modifying membrane integrity [254]. Also, covalent/non-covalent modification with charged groups changes surface membrane charge and directly contributes to the internalization of the EVs to the target cells [254] and also may determine how long EVs can remain in the systemic circulation. For example, while incorporation of covalent chains of polyethylene glycol (PEGylation) into the membrane of EVs does prolong their intact-state circulatory life, this is accompanied by T cell-dependent activation against PEG-containing vesicles that destroy the vesicles [255]. In turn, the excessive activation of immune cells represents a great challenge that requires careful evaluation of potential immune-related risks when chemically modified EVs are introduced to the organism. However, in anti-tumor activity the functionalization of EVs by hydrophobic modification by cholesterol or PEG conjugated with a molecule with a certain cytotoxic effect or by electrostatic insertion of a functional molecule has shown its effectiveness [256,257]. Despite the above-mentioned limitations and potential issues with EVs stability and toxicity, facilitation of the loading by modified EVs properties represents a highly effective way for repletion of EVs with a target cargo, including various RNAs. The next section in our discussion relates to the molecular mechanisms by which RNA-preloaded EVs can modify, regulate, or inhibit inflammatory signaling and immune cell behavior in various autoimmune diseases.

## 3. Mechanistic Foundation of Extracellular Vesicles as Immune Information Carriers

A comprehensive understanding of the molecular mechanisms of the pathogenesis of an autoimmune disease is necessary to target this disease process more precisely and with predicted outcomes. The plethora of autoimmune disorders includes those initiated/mediated by various mechanisms. We employed current findings about key effector cells, dominant intra- and intercellular mechanisms, and target cells involved in the pathogenesis of the most common autoimmune disorders. We split the disorders into two large categories based on the effect on the body: systemic and organ-specific. The systemic autoimmune disorders include systemic lupus erythematosus, rheumatoid arthritis, Sjögren’s syndrome, and systemic sclerosis. A much larger subset of organ-specific autoimmune disorders includes inflammatory bowel disease and colitis, autoimmune hepatitis, multiple sclerosis, psoriasis, type 1 diabetes mellitus, Graves’ disease, and myasthenia gravis, etc. (only those listed in Table 2 are mentioned here). Table 2 represents distilled information about these disorders, and one could see that the underlying molecular players and exact mechanisms differ significantly between these pathologies. For example, despite the fact that systemic lupus erythematosus and rheumatoid arthritis share their strong systemic influence and both have genetic predisposition factors, these pathological states are based on distinct pathways of pathogenesis: impaired clearance of dying/died cells and provoked and looped autoantibody production by B cells vs. hyperactivity in proliferation and producing immune cytokines by fibroblast-like synoviocytes (FLS) resulting in metalloprotease-mediated destruction of collagen type II in various tissues.

The variety of the pathological pathways and their embedded specific components should be addressed in the development of therapy based on targeted delivery of RNA-preloaded EVs (or other therapies as well). Having precise knowledge of molecular targets and target cells is ultimately important for further selection of miRNA(s) which can be effectively loaded into EVs and subsequently alter the pathogenic states, as well as result in immune tolerance and functional recovery of affected tissues, organs, and systems. EVs regulate the immune system by acting as intercellular messengers that transfer bioactive cargoes between cells. Via the delivery of RNA species, EVs are capable of modulating innate and adaptive immune responses, regulating inflammatory signaling, and promoting or disrupting immune tolerance. EV-based RNA delivery allows cells to undergo post-transcriptional and epigenetic reprogramming, establishing fine-tuning of immune activation or suppression in autoimmune diseases. In the following section, we outline core mechanisms of action of EVs.

### 3.1. Core Molecular Pathways Regulated by RNA-Loaded Extracellular Vesicles

#### 3.1.1. Inflammasome Regulation and Pyroptosis

Inflammasomes are multiprotein complexes that promote inflammation by triggering caspase-1 to release inflammatory cytokines (IL-1β, IL-18). Aberrant or dysregulated activation of inflammasomes contributes to the progression and pathogenesis of autoimmune diseases [270]. EV-delivered non-coding RNAs, particularly miRNAs, are essential for controlling inflammasome activity by altering gene expression through translational repression or post-transcriptional modification [270]. While broadband suppression of NF-κB and STAT3 pathways provides systemic relief from inflammation [271,272], more targeted approaches address the NLR family pyrin domain-containing 3 (NLRP3) inflammasome, the primary source of IL-1β cytokine storms.

The RNA-related cargo in exosomes and EVs can activate NLRP3 via canonical and non-canonical ways [273]. In the canonical way, the establishment of NLRP3 inflammasome requires initial activation of NF-κB pathway via TLR ligands or damage-associated/pathogen-associated molecular patterns (DAMPs/PAMPs) followed by exposing the cells to certain molecular factors acting as a danger signal, i.e., this is a multi-step process. The whole cascade is completed by activation of caspase-1, which converts inactive forms of intracellular pro-inflammatory cytokines to their respective active forms and promotes their release into extracellular space. Accordingly, loading of EVs by miRNAs that could interact with these cascades has prominent potential to control the activation and, more importantly in autoimmune disorders, inhibition of NLRP3 inflammasome. On the other hand, indirect mechanisms can also result in the activation of NLRP3 via non-canonical ways. As an example, EVs cargoed by miR-148a can directly regulate function of Gasdermin D, which is the protein that forms pores in the lipid membrane. Poring the membrane facilitates efflux of potassium and contributes to the activation of NLRP3 inflammasome [273]. Interestingly, this process is managed by a positive feedback loop, so potassium efflux-induced elevation of NLRP3 inflammasomes further perforates the cell membrane and finally results in pyroptotic cell death [274,275]. Therefore, targeting the activation of NLRP3 inflammasome by RNA-loaded EVs that lead to the pyroptotic mechanism of cell death should consider these self-augmenting processes to avoid the overrunning of cell lysis (i.e., excessive triggering in many cells).

In the context of autoimmune diseases, the beneficial effect of EV-based therapy must be based on inhibition of NLRP3 inflammasome and related counterparts. Several studies demonstrate that EV-encapsulated miRNAs directly inhibit NLRP3 inflammasome activation. miR-233 delivered by BMSC-derived EVs to recipient cells directly targets and inhibits the NLRP3 gene, preventing NLRP3 inflammasome assembly and decreasing inflammatory cytokine secretion, including interleukin-1β (IL-1β), tumor necrosis factor α (TNF-α), and interleukin-18 (IL-18) [199]. A similar mechanism has been described for liver protection in experimental autoimmune hepatitis [193]. Furthermore, in rheumatoid arthritis, BMSC-derived EVs carrying miR-515-5p specifically target the TLR4/NLRP3/GSDMD axis in fibroblast-like synoviocytes (FLS). By silencing TLR4, not only does it prevent the assembly of the NLRP3 inflammasome but also preserves mitochondrial integrity and blocks the pore-forming activity of Gasdermin D. Consequently, this averts the pyroptotic cell death and release of high concentrations of pro-inflammatory cytokines [198]. In colitis, the symptoms were relieved by miR-203a-3p.2-loaded small EVs from human UCMSCs by inhibiting CASP11/4, decreasing IL-1β, IL-6, and casp11 secretion, and reducing macrophage pyroptosis in dextran sulfate sodium (DSS)-induced mouse inflammatory bowel disease [276]. miRNAs derived from small EVs in the plasma of SLE patients specifically target TLR7 and TLR8 signaling pathways, thereby inhibiting autoimmune-mediated inflammation in SLE [277].

**Figure 1:**
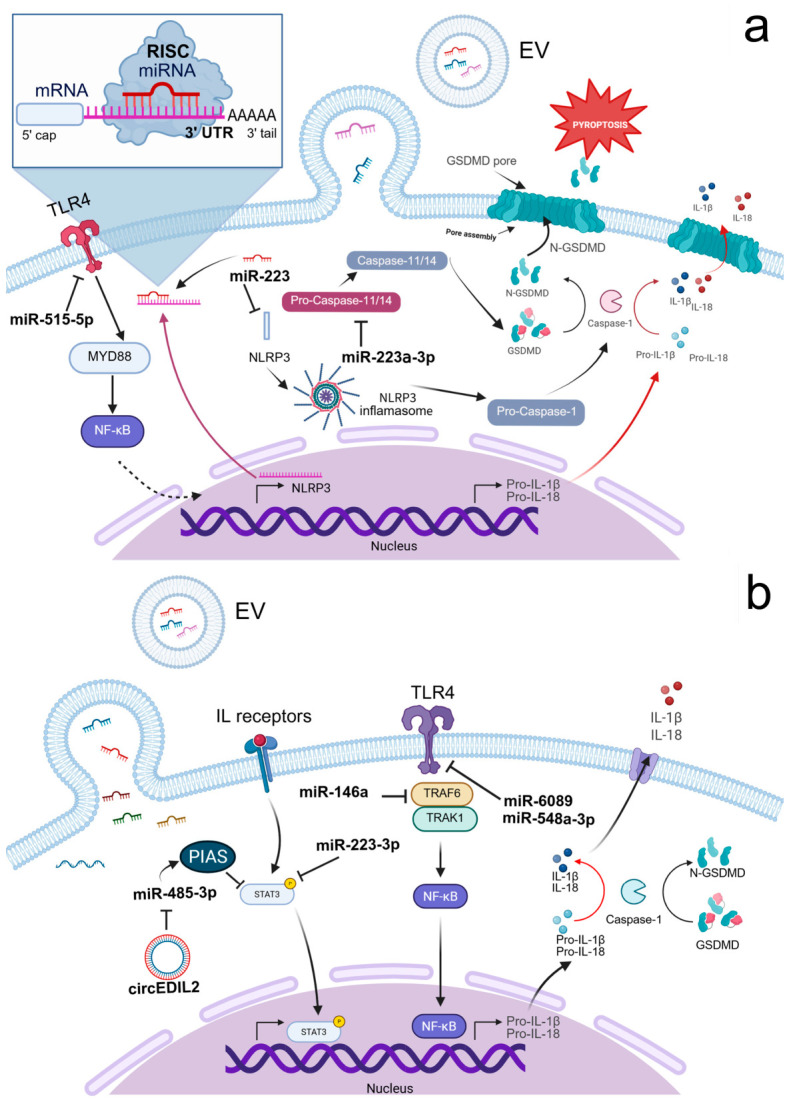
EV-derived miRNAs regulate inflammatory signaling and pyroptosis. (Panel (**a**)) Regulation of the NLRP3 inflammasome and pyroptosis. Extracellular vesicles (EVs) deliver miRNAs that modulate the priming and activation of the inflammasome. TLR4 activation triggers the MYD88/NF-κB pathway, inducing transcription of NLRP3, Pro-IL-1β, and Pro-IL-18. This priming is inhibited by miRNA-515-5p and miR-223. Caspase-1, activated by the NLRP3 inflammasome, cleaves pro-cytokines into mature IL-1β and IL-18. Additionally, miRNA-223a-3p targets Pro-Caspase-11/14. Active caspases cleave GSDMD into N-GSDMD, which forms membrane pores leading to pyroptosis. The inset in Panel (**a**) shows schematically how miRNA is interacting with target mRNA. (Panel (**b**)) Integration of EV-cargo in TLR4 and STAT3 signaling. EVs modulate cellular responses via multiple RNA species. The TLR4/NF-κB axis is suppressed by miR-6089, miR-548a-3p, and miR-146a (targeting TRAF6). Simultaneously, the STAT3 pathway is regulated by miR-223-3p and miR-485-3p, the latter being modulated by circEDIL2 and PIAS. EV-mediated delivery of IL-10 mRNA further promotes anti-inflammatory action, counteracting pro-inflammatory cytokine production. The image was created in Biorender by Aliya Orassay and Naizabek Yerzhigit (2026), https://app.biorender.com/illustrations/695f3633c3e85e4865498907?slideId=809bb64f-8cbc-409b-8a86-a1d05c7ec601 (accessed on 12 April 2026) for Panel (**a**) and https://app.biorender.com/illustrations/695f3633c3e85e4865498907?slideId=8b9e5e77-0a0d-4860-a53e-682dc4ebd9a6 (accessed on 12 April 2026) for Panel (**b**).

Figure 1 illustrates the key pathways involved in NLRP3 inflammasome and pyroptosis, as well as in TLR4 and STAT3 signaling. These pathways can be stimulated upon the release of exosomal miRNAs directly into a cell, and several cascades could be activated via EV due to its highly heterogeneous content.

#### 3.1.2. Autophagy–Inflammation Crosstalk

Autophagy and its core regulatory proteins play a decisive role in the development of autoimmune diseases. Recent studies have shown that autophagy assists innate immunity by pathogen clearance and inflammation regulation [278]. One of the core mechanisms of inflammation suppression is achieved by regulating inflammasome activation, including the NLRP3 inflammasome [152,153].

Dysregulation of autophagy-regulated genes results in the pathogenesis of autoimmune diseases by promoting chronic inflammation and loss of immune tolerance [279]. For instance, autophagy-related 5 protein (ATG5) is critical to initiate SLE pathogenesis. Beyond its role in degradation, ATG5 is essential for B-cell receptor polarization and centrosome relocalization to the immune synapse. This autophagy-mediated process supports the survival of autoreactive B cells and is mandatory for their differentiation into plasmablasts, thereby triggering the onset of clinical SLE [280,281].

miRNAs, collectively known as ‘autophagomirs,’ establish a sophisticated post-transcriptional regulatory network that orchestrates every phase of the autophagy pathway by targeting core autophagy-related genes and upstream signaling nodes [278]. As an example, EVs derived from genetically modified ADSCs overexpressing miR-20a exert potent therapeutic effects in murine lupus nephritis.

In the case of systemic sclerosis, Chen et al. (2017) demonstrated that EVs containing miR-151-5p derived from MSCs of donors can reduce osteopenia in a mouse model of systemic sclerosis [196]. These EVs carry miR-20a to the kidneys, where it interferes with the Akt/mTOR pathway, ensuring the smooth functioning of autophagy and protecting podocytes from immune-mediated damage [194]. This is mediated through IL4Rα inhibition, which in turn reduces mTOR signaling in the recipient BMSCs [196].

However, the role of autophagy appears to depend on the specific context. While some studies have aimed to enhance autophagy to improve cell survival, other studies have demonstrated the role of EVs in maintaining vascular integrity in systemic sclerosis by inhibiting excessive autophagy. For example, Zhu et al. (2024) demonstrated that EVs containing miR-126-3p have protective effects against vascular damage through interaction with SLC7A5 [201]. This interaction with mTOR signaling in endothelial cells reduces autophagy-related damage and enhances angiogenesis, providing a preventive strategy for microangiopathy in early progression of systemic sclerosis.

Beyond miRNAs, long non-coding RNAs (lncRNAs) have emerged as pivotal regulators of complex autoimmune phenotypes. For instance, lncRNA small nucleolar RNA host gene 16 (SNHG16) has been identified as a key driver of TLR4-mediated autophagy and NETosis in the lungs of mice and patients with SLE-associated alveolar hemorrhage. Experimental evidence suggests that targeting SNHG16 using shRNA can effectively suppress these aberrant immune responses, offering a potential therapeutic strategy for mitigating alveolar hemorrhage associated with SLE [282].

#### 3.1.3. NF-κB, STAT3, and MAPK Inflammatory Signaling

RNA-loaded EVs modulate key inflammatory signaling cascades, including NF-κB, STAT3, and MAPK pathways, which are central drivers of autoimmune inflammation. Suppression of these pathways results in decreased cytokine production and attenuation of tissue damage.

In experimental colitis, EV-encapsulated miR-146a suppresses innate immune signaling by directly targeting the adaptor proteins TRAF6 and IRAK1, thereby inhibiting NF-κB activation. This downstream attenuation of NF-κB signaling leads to reduced production of pro-inflammatory cytokines, including TNF-α, IL-6, and IL-1β, ultimately ameliorating intestinal inflammation [283]. Inhibition of pro-inflammatory and blockade of the NF-κB pathway can be achieved by delivery of anti-inflammatory cytokines. For instance, delivery of IL-10 mRNA via EVs had a potent anti-inflammatory effect in the inflammatory bowel disease mouse model [150]. Other studies suggested that the delivery of EV-encapsulated miR-204-3p, miR-7977, miR-1237-5p, miR-5787, and miR-6089 may inhibit transcription of fibrosis-related genes in target cells (e.g. hepatic stellate cells) via modulation of activity of small GTPase proteins that directly contribute to the protection from excessive fibrosis in the site of inflammation [284]. Similarly, Wang et al. (2019) demonstrated that miR-548a-3p downregulates the inflammatory TLR4/NF-κB signaling cascade in rheumatoid arthritis, reinforcing the role of TLR4 as a mechanistic hub targeted by RNA-loaded EVs in autoimmune inflammation [285].

While miRNAs like miR-124a and miR-223-3p focus on suppressing overactive inflammatory cascades, other regulators, such as miR-146b, work to actively repair the site of injury. Specifically, miR-146b targets Siah2 to stabilize TRAF proteins, ensuring that the NF-κB-dependent survival and barrier function of intestinal epithelial cells remain intact during autoimmune-like insults [286].

Targeting the STAT3 signaling pathway is another key target for RNA-loaded EV-based therapy. MSC-derived EVs carrying miR-223-3p present immunoregulation through modulating STAT3 signaling, which results in a rebalancing of the Treg/Th17 axis and a decrease in the expression of pro-inflammatory cytokines IL-1β and IL-6 [286]. This strategy effectively reduced liver injury by decreasing serum levels of ALT and AST, improving histological scores, and mitigating inflammatory lesions in the liver [195]. The study on the animal model of collagen-induced arthritis showed that engineered circEDIL3 EVs from synovial MSCs upon their transfer to rheumatoid arthritis FLS reduce inflammation-driven angiogenesis. It targets miR-485-3p, which acts on the PIAS3 protein inhibitor responsible for STAT3 suppression [210].

Moreover, Xie et al. (2023) showed that inhibiting the IL-33/ST2 axis by BMSC-derived EVs delivering miRNA-214 alleviates skin fibrosis in systemic sclerosis [287]. IL-33/ST2 axis contributes to upregulation of pro-inflammatory cytokines, suggesting thatthe inhibition of this signaling pathway prevents autoimmune diseases [288]. Other studies based on the rheumatoid arthritis model in rats demonstrate suppression of proliferation, migration, and inflammation by exosomal circFBXW7 in rheumatoid arthritis FLS, which are producers of pro-inflammatory cytokines and matrix-degrading enzymes in rheumatoid arthritis. This circFBXW7 upregulates the Histone Deacetylase 4 (HDAC4) expression by directly acting as a sponge for miR-216a-3p [209].

### 3.2. Cellular Reprogramming of Autoimmunity by RNA-Loaded Extracellular Vesicles

#### 3.2.1. Macrophage Polarization (M1-M2 Axis)

**Figure 2:**
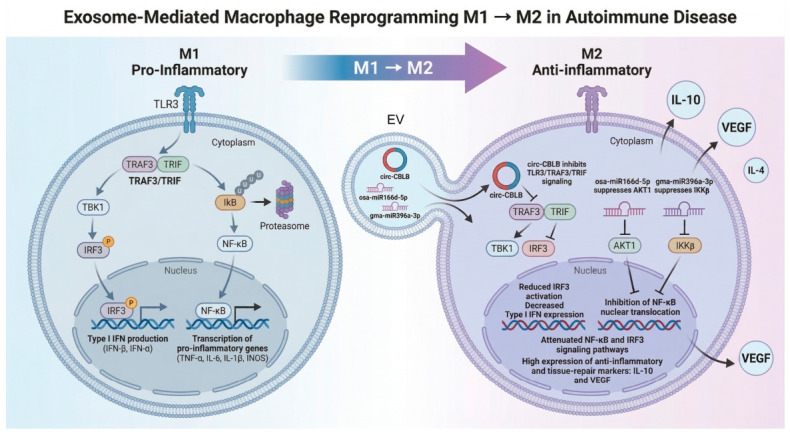
EV-mediated macrophage reprogramming from M1 to M2 phenotype. EVs drive the phenotypic transition of macrophages from a pro-inflammatory (M1) to an anti-inflammatory (M2) state in autoimmune contexts. In the M1 state, TLR3 signaling through the TRAF3/TRIF complex activates TBK1/IRF3 and NF-κB pathways, inducing the production of Type I IFNs and pro-inflammatory mediators such as TNF-α, IL-6, and iNOS. Exosomal delivery of circ-CBLB, osa-miR166d-5p, and gma-miR396a-3p interrupts this axis by inhibiting the TLR3/TRAF3/TRIF complex, AKT1, and IKKβ, respectively. This intervention attenuates IRF3 and NF-κB signaling, thereby reprogramming the cell toward an M2 phenotype characterized by the secretion of IL-10, VEGF, and IL-4, promoting tissue repair. Arrows crossing the plasma membrane show the secretion of factors/cytokines by the cell to the extracellular environment. Arrows/inhibiting lines inside the cell show appropriate signaling pathways. Arrows arising from DNA indicate transcription of corresponding genes. Created using Nano Banana Pro (Google Gemini) with further manual editing.

Macrophage polarization is a critical determinant of the progression of many autoimmune diseases [289]. An imbalance between M1 and M2 macrophage phenotypes plays a pivotal role in the pathogenesis of autoimmune diseases. Most studies indicate that increasing the prevalence of M2 macrophages and promoting an anti-inflammatory phenotype alleviates disease severity [290,291,292,293]. It has been shown that EVs exert immunomodulatory function by suppressing M1 macrophage activation and promoting M2 macrophage polarization. This indirectly supports mechanisms of Treg cell formation and reduces autoimmune reactions by developing immune tolerance, highlighting their potential as a cell-free therapeutic strategy [294]. The schematic representation of key signaling cascades that are mediated by the uptake of EVs by macrophages and drive M1-to-M2 transition is shown in Figure 2.

Notably, non-coding RNAs can regulate macrophage polarization by either promoting pro-inflammatory M1 phenotypes or driving anti-inflammatory M2 polarization [295,296]. For example, in the research by Giri et al. (2024) EL-4 T cell-derived EVs carrying miR-155 were delivered to EL-4 cells [297]. As a result, an abundance of miR-155 led to the downregulation of genes including Tea-shirt Zinc Finger Homeobox 3 (TSHZ3), Jumonji AT-rich interactive domain 2 (Jarid2), zinc finger protein 652 (ZFP652), and WW domain-containing protein 1 (WWC1). Further analysis demonstrated that these micro EVs (mEVs) promoted polarization of macrophages toward an M1 phenotype, reflected by elevated TNF-α, IL-6, IL-1β, and iNOS expression [297]. Other studies have demonstrated that the expression of circ-CBLB in the EVs from rheumatoid arthritis patients was lower, which showed a correlation with stronger activation of the TLR3/TRAF3 signaling axis and M1 macrophage polarization. On the contrary, circ-CBLB-rich EV-based treatment of macrophages suppressed the activation of the TLR3/TRIF/TRAF3 pathway, attenuating the transformation of M0 to M1 and suppressing the expression of inflammatory factors in rheumatoid arthritis patients [298].

#### 3.2.2. Regulation of Fibroblast-like Synoviocytes

The process of hyper-proliferation and invasion by fibroblast-like synoviocytes (FLSs) causes joint destruction in rheumatoid arthritis. RNA-loaded EVs target synoviocytes in a direct manner and affect their proliferation, survival, and inflammatory processes. To correct the abnormal microenvironment in tissues, MSC EVs target the abnormal overgrowth of FLS. In particular, EVs overexpressing miRNA-124a efficiently suppress FLS proliferation and trigger G0/G1 cell cycle arrest, a crucial mechanism that directly opposes the aggressive synovial hyperplasia typical of rheumatoid arthritis [191]. Another miRNA, miR-338-5p, has been shown to be overexpressed in the FLS in rheumatoid arthritis [299]. It was found that this miRNA regulates proliferative activity and migration of FLS via nuclear factor of activated T cells 5 (NFAT5), which directly suppresses FLS proliferation. Of note, NFAT5 represents a factor with multiple functions on immune cells including macrophages, monocytes, and T cells, and it is a crucial player in autoimmune pathogenesis [300]. For example, in patients with active forms of vitiligo, the underexpression of NFAT5, alongside deficient expression of FoxP3, is linked to the dysregulated activity of Tregs [301]. The same was reported in type 1 diabetes mellitus, where unbalanced NFAT5 function was linked to impaired differentiation of Tregs and elevated autoimmune aggression against pancreatic islets [300]. In addition, overexpression of miR-338-5p in BMSC-derived EVs protects cells from excessive apoptosis induced by the PI3K/Akt pathway under oxidative stress [302]. Similarly, Mi et al. (2024) demonstrated that human UCMSC-derived EVs loaded with miR-451a specifically target activating transcription factor 2 (ATF2) in synovial fibroblasts in rheumatoid arthritis [170]. This delivery effectively degrades ATF2 transcripts, leading to the inhibition of the aggressive ‘tumor-like’ proliferation, migration, and invasive capacity of the synoviocytes, thereby reducing joint destruction.

Beyond direct control of synoviocyte proliferation, RNA-loaded EVs modulate angiogenesis and matrix remodeling. Chen et al. (2018) revealed the therapeutic potential of MSC-derived miR-150-5p small EVs in rheumatoid arthritis [157]. The miR-150-5p targets MMP14 and VEGF by binding to 3′-UTRs and suppressing their expression, subsequently performing an anti-inflammatory effect and inhibiting angiogenesis in vivo, which serves as a hallmark of rheumatoid arthritis progression. Complementarily, MSC-derived EVs loaded with miR-320a inhibit the progression of rheumatoid arthritis by targeting CXCL9 and subsequently reducing expression of immune factors (IL-1, IL-6, IL-8) [160].

In addition to targeting chemokines, EV-encapsulated miRNAs can reprogram the signaling architecture of synovial fibroblasts. At the intracellular signaling level, EVs from chondrogenically differentiated BMSCs carry high levels of miR-205-5p, which targets mouse double minute 2 homolog (MDM2). By inhibiting MDM2-induced signaling of the MAPK and NF-κB pathways, these EVs reduce the expression of matrix metalloproteinases and inflammatory cytokines, indicating a dual mechanism that not only suppresses but also protects joint tissues from degradation in the models of rheumatoid arthritis [203].

Similarly, the delivery of miR-140-3p via human UCMSC-derived EVs targets serum- and glucocorticoid-regulated kinase 1 (SGK1), which in turn triggers apoptosis in synovial fibroblasts of rheumatoid arthritis patients. This is highlighted by reduced secretion of TNF-α and IL-1β, indicating a shutdown of the inflammatory feedback loop. This molecular targeting not only stops the migration and proliferation of synovial fibroblasts but also minimizes oxidative stress and fibrosis, indicating a move away from immunosuppression and towards tissue repair [199].

**Figure 3:**
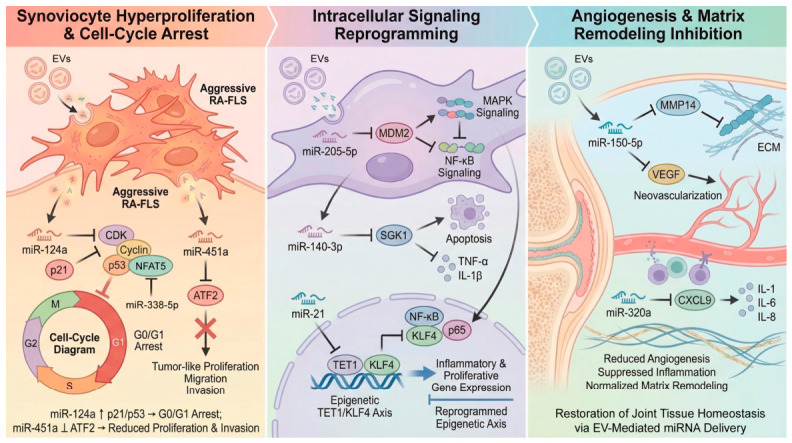
Mesenchymal stem cell (MSC)-derived EV-mediated therapeutic mechanisms in rheumatoid arthritis. MSC-derived EVs restore joint tissue homeostasis in rheumatoid arthritis (RA) through three primary integrated mechanisms. First, they counteract synoviocyte hyperproliferation and invasion by delivering miR-124a or miR-338-5p, which trigger G0/G1 cell cycle arrest (via different mediators), and miR-451a, which targets ATF2 transcripts to inhibit the aggressive “tumor-like” phenotype of RA-FLS. Second, EV cargo reprograms intracellular signaling: miR-205-5p suppresses MDM2 to inhibit MAPK and NF-κB pathways, while miR-140-3p targets SGK1 to induce apoptosis and reduce TNF-α and IL-1β levels. Additionally, miR-21 targets the TET1/KLF4 axis to epigenetically shift FLSs toward a non-pathological state. Finally, MSC EVs inhibit angiogenesis and extracellular matrix (ECM) remodeling through miR-150-5p-mediated suppression of MMP14 and VEGF, alongside miR-320a targeting of CXCL9 to reduce pro-inflammatory cytokine expression. Arrows/inhibiting lines show appropriate signaling pathways. Created using Nano Banana Pro (Google Gemini) with further manual editing.

Figure 3 summarizes how the above-mentioned mechanisms in rheumatoid arthritis can be mediated by EV uptake that results in the activation or inhibition of target cascades producing an intra/extracellular environment favorable to induce apoptosis of hyper-proliferative FLSs (left panel on the illustration), to reprogram immune cells to anti-inflammatory profile (middle panel), and to prevent angiogenesis and pathological extracellular matrix remodeling (right panel).

Rheumatoid arthritis joint remodeling is also influenced by EV-mediated osteo-immune communication. For example, EV-encapsulated miR-486-5p enhances osteoblast differentiation through the inhibition of Tob1 and activation of the BMP/Smad pathway, which is associated with a milder form of arthritis in a collagen-induced arthritis model. Thus, EV-encapsulated miR-486-5p secreted by rheumatoid arthritis FLS emerges as a therapeutic target for the treatment of rheumatoid arthritis [303]. Moreover, Li et al. (2021) have established that EVs secreted by mesenchymal stem cells of bone marrow origin deliver miR-21 to synovial fibroblasts [158]. Once inside the FLSs, miR-21 targets the TET1/KLF4 axis by regulating the extent of TET1-induced DNA hydroxymethylation of the KLF4 promoter. This epigenetic reprogramming of synovial fibroblasts suppresses their aberrant proliferation and inflammatory responses in models of rheumatoid arthritis.

### 3.3. Restoration of Immune Tolerance

#### 3.3.1. Induction of Tolerogenic Antigen-Presenting Cells

Restoring immune tolerance is a central therapeutic objective in autoimmune diseases. The pathogenesis of autoimmune diseases is mediated through self-reactive T cells that escape immune tolerance and induce self-tissue damage, assisting B cells to initiate autoantibody production. Enhancing immune tolerance indeed appears to be a promising approach for the treatment of autoimmune diseases. Among approaches, one involves the induction of tolerogenic antigen-presenting cells (APCs) that negatively regulate the activation of autoreactive T cells by inducing T cell anergy, wherein T cells are functionally nonresponsive and cannot proliferate or produce IL-2, promoting differentiation of Treg cells, and eliminating autoreactive T cells [304,305,306].

Dendritic cell (DC) activation state determines immunogenic versus tolerogenic function. While mature DCs are responsible for generating effector T cells, tolerogenic DCs originate from immature, semi-mature, or mature DCs and lead to T cell anergy, Tregs, and tolerance [307]. These tolerogenic DCs are regulated through specific transcriptional profiles and signals such as IL-10, TGFβ, and aryl hydrocarbon receptor (AHR) signaling [308,309], microbial metabolites [310], and the uptake of apoptotic cells [311] that downregulate co-stimulation and inflammatory cytokines while promoting anti-inflammatory mediators, offering therapeutic opportunities for immune modulation [305].

Emerging evidence indicates that RNA cargo delivered by EVs plays a critical role in programming tolerogenic APC function. While earlier studies on MSC-derived EVs emphasized surface-bound proteins such as programmed death protein ligand 1 (PD-L1) and TGF-β in suppressing autoreactive lymphocyte proliferation and promoting Treg expansion [312], more recent work supports a direct immunomodulatory role for RNA-encoded EVs. For instance, Liu et al. (2025) showed that delivery of mRNA coding PDL1 via lipid nanoparticles (LNPs) can generate tolerogenic APCs in the mouse model of rheumatoid arthritis and ulcerative colitis [313]. The delivery of PD-L1 to APCs by LNPs alleviated disease progression by producing a tolerogenic phenotype, which subsequently targets activated T cells and expands the Treg cell population. mRNA encapsulated with LNPs enters the cells and is translated to antigens or epitopes in antigen-presenting cells to form immune tolerance, by training the immune system to recognize the epitope and produce Treg cells, which suppresses excessive immune responses towards self-antigens [314].

Consistent with this concept, EV-associated miRNAs have been shown to modulate DC maturation and function. MSC-derived EVs suppress antigen uptake by immature DCs, interfering with DC maturation and function, decreasing pro-inflammatory cytokine production, and promoting anti-inflammatory cytokine secretion. Particularly, MSC-EVs are enriched in DC-regulatory miRNAs, including miR-21-5p, which targets C-C motif chemokine receptor 7 (CCR7); DCs transfected with miR-21-5p showed reduced CCR7 expression and impaired migration toward CCL21. These findings indicate that MSC-EV-derived miRNAs can recapitulate MSC-mediated DC modulation, supporting MSC-EVs as a potential cell-free immunomodulatory therapy [315]. There is another pathway that utilizes direct incorporation of an antigenic peptide carried by EV into the surface of APCs via cross-dressing mechanism [36]. This EV-mediated modification of the antigen presentation by APC represents a relatively novel approach which needs to be further verified in preclinical and clinical studies. The immune tolerance can also be experimentally achieved in vitro or in vivo in mice by systemic administration (intravenously or orally) of small EVs containing miRNA-150, specific antigens, and pre-coated by the antibody-free light chains [316]. It is also noteworthy that some researchers precondition APCs like DC and macrophages with specific self-antigens to generate immune-tolerant EVs—while this mechanism does not imply direct transfer of RNAs from EVs to target cells, it still utilizes specific RNAs involved into translation of the self-antigens [317].

#### 3.3.2. Th17/Treg Axis Rebalancing, Induction and Expansion of Regulatory T Cells

An imbalance between pro-inflammatory Th17 cells and regulatory T cells (Tregs) is a defining feature of many autoimmune diseases. Thus, restoration of immune tolerance remains critically dependent on the correction of the Th17/Treg imbalance. Current studies have shown that engineered EVs can regulate the Th17/Treg axis through the delivery of specific RNAs. This can occur either through the inhibition of pathologic Th17 cell differentiation or through the promotion of the generation and expansion of regulatory T cell populations.

A second method of immune rebalancing can occur through the direct inhibition of Th17 cell differentiation. Xie et al. (2025) found that engineered EVs carrying the miRNA let-7f-5p can suppress Th17 cells through the inhibition of the RORC/IL-17A pathway to restore immune homeostasis [167]. This method represents a new avenue for immune tolerance restoration in autoimmune diseases through the direct targeting of pathologic Th17 cells via the inhibition of RORC, the key transcription factor controlling Th17 cell differentiation [318]. Similarly, miRNA-223-3p can regulate immune homeostasis through the enhancement of the Treg/Th17 cell ratio to restore immune homeostasis and suppress inflammation in autoimmune hepatitis and thus represents a conserved mechanism of immune regulation via the restraint of Th17 cell-mediated pathology across disease contexts [195].

At the same time, RNA-loaded EVs can also promote the generation and expansion of regulatory T cells. Tu et al. (2022) indicate that UCMSC-derived EVs are more enriched with miR-19b compared to UCMSCs, and transduction of the RNA content into CD4+ cells increases the miR-19b level in cells, consequently increasing the ratio of Treg cells, and reducing the ratio of Th17 cells in SLE patients [200]. Consistently, MSC-derived EVs enriched with miR-20a-5p were shown to reinstate their capacity to facilitate Treg development and therapeutic efficacy in SLE models [319]. Recently, the antigen-presenting EVs (AP-EVs) that co-display peptide-major histocompatibility complex class II complexes (pMHCII), interleukin-2 (IL-2), and transforming growth factor-β (TGF-β) on their surface have been used. These immunomodulatory molecules were anchored to the EV membrane via CD81 or milk fat globule-EGF factor 8 (MFG-E8) scaffolds to ensure stable and multivalent presentation. AP-EVs induced the differentiation of antigen-specific Tregs from naïve CD4^+^ T cells in vitro, and promoted their proliferation and expression of canonical regulatory markers, including CD25, CTLA-4, PD-L1, and LAG-3. In vivo, the combination of AP-EVs and mTOR inhibition with rapamycin significantly enhanced the generation of FoxP3^+^ Tregs in antigen-specific adoptive transfer models. The Tregs induced by AP-EVs in vitro exhibited suppressive function [320].

At the signaling level, RNA-loaded EVs modulate intracellular pathways that favor Treg lineage commitment. Chen et al. (2024) reported that miRNA-148a encapsulated with gingival MSC-EVs after uptake by T cells targets IKKβ and silences the NF-κB signaling pathway [204]. This molecular intervention creates a permissive environment for FoxP3+ Treg differentiation while simultaneously antagonizing the Th17 programming. Complementarily, MSC-derived EVs enriched with lncRNA TUG1 deliver this cargo to CD4^+^ T cells, where TUG1 upregulates BLIMP1 expression, thereby modulating the Th17/Treg cell balance, reducing pathogenic Th17 cells while increasing regulatory T cells, and ultimately alleviating rheumatoid arthritis-related joint damage, highlighting their potential as a therapeutic strategy for rheumatoid arthritis [321]. Among all highly expressed miRNAs in EVs derived from TGF-β-induced Treg cells, miR-449a-5p demonstrated modulation of target Notch1 expression, reducing Th17 cell frequency and shifting towards Treg cells in arthritic mice [322].

#### 3.3.3. Th17-Independent Regulation of T Cells

Beyond Th17 suppression, EV-mediated RNA transfer directly supports peripheral immune tolerance by inducing and expanding regulatory T cell subsets, including FoxP3^+^ Tregs and IL-10-producing Tr1 cells. Xiong et al. demonstrated that human placenta MSC-derived EVs enriched with miR-21 protect CD4+ T cells from aging-related oxidative damage. By targeting the PTEN/PI3K-Nrf2 axis, these EVs reduce the accumulation of reactive oxygen species (ROS) and inhibit apoptosis, thereby preserving a functional T cell pool and preventing the pro-inflammatory decline typically associated with chronic autoimmune progression [323].

RNA-loaded EVs also indirectly promote Treg expansion through modulation of antigen-presenting cells. Yin et al. (2017) showed that EVs derived from miR-146a overexpressing dendritic cells (DCs) decrease CD80 and CD86 levels [324]. Treatment alleviates the clinical symptoms of experimental autoimmune myasthenia gravis in mice, decreases serum anti-AChR IgG, IgG1, and IgG2b levels, and alters the T helper cell cytokine profiles from Th1/Th17 to Th2/Treg cells in serum and spleen. In collagen-induced arthritis models, miR-146a transduced MSC-derived EVs were shown to increase the expression of Fox-P3, TGF-β, and IL-10 in vivo. Moreover, EVs carrying miR-155 showed upregulation of RORγt, IL-17, and IL-6 genes. An increase in Treg cell populations was observed, highlighting the therapeutic effect of RNA-loaded EVs [192].

Microvesicles derived from human MSCs have also been shown to regulate T cell responses in patients with type 1 diabetes. Upon internalization of EVs by peripheral blood mononuclear cells, the main effect of antigen-driven T cell activation (CD4 + IL-10+ Tr1 cell) and induction of Treg phenotype (CD4 + CD25 + FoxP3+ Treg) was observed. When T cells interact with microvesicles, the TGFB1 transcript level was increased, which can also be attributed to the transfer of TGFB1 mRNA [325]. Similarly, the effect of miR-14 on TGF-β induction was elucidated [326].

Further evidence highlights disease-specific applications of RNA-loaded EVs to regulate immune tolerance. Wang et al. (2024) demonstrated that human UCMSC-derived EVs effectively relieve vitiligo by delivering miR-132-3p and miR-125b-5p, which target Sirt1 and Bak1, respectively [327]. Consequently, this leads to decreased CD8+ T cell infiltration and activated Treg cell-mediated immune suppression.

#### 3.3.4. EV-Mediated miRNA Regulation of Stress-Induced Cell Death and Fibrotic Remodeling

Furthermore, the therapeutic potential of MSC-derived EVs is not only restricted to the peripheral immune compartment but also extends to other anatomically and immunologically specialized tissues such as the central nervous system and fibrotic mesenchymal tissues. In the context of the central nervous system, Fan et al. (2023) showed the therapeutic potential of MSCs-derived EVs carrying miR-367-3p in inhibiting microglial ferroptosis through the downregulation of the expression of the Enhancer of zeste homolog 2 (EZH2) and the upregulation of the expression of the solute carrier family 7 member 11 (SLC7A11) in the context of the central nervous system [197]. Additionally, the symptom relief was observed using the experimental autoimmune encephalomyelitis model, which is a model for multiple sclerosis. In addition to the modulation of the immune system in the central nervous system, the therapeutic capacity of MSC-derived EVs extends to the modulation of fibrotic tissue niches. The therapeutic effects of the delivery of antifibrotic miRNA-196b-5p using MSC-derived EVs on the suppression of the expression of collagen type I in dermal fibroblasts and the attenuation of fibrosis in the context of systemic sclerosis have also been explored [205]. Together, these studies highlight the versatility of MSC-derived EVs as RNA delivery platforms capable of modulating diverse pathological microenvironments, despite disease- and tissue-specific mechanisms of action.

**Figure 4:**
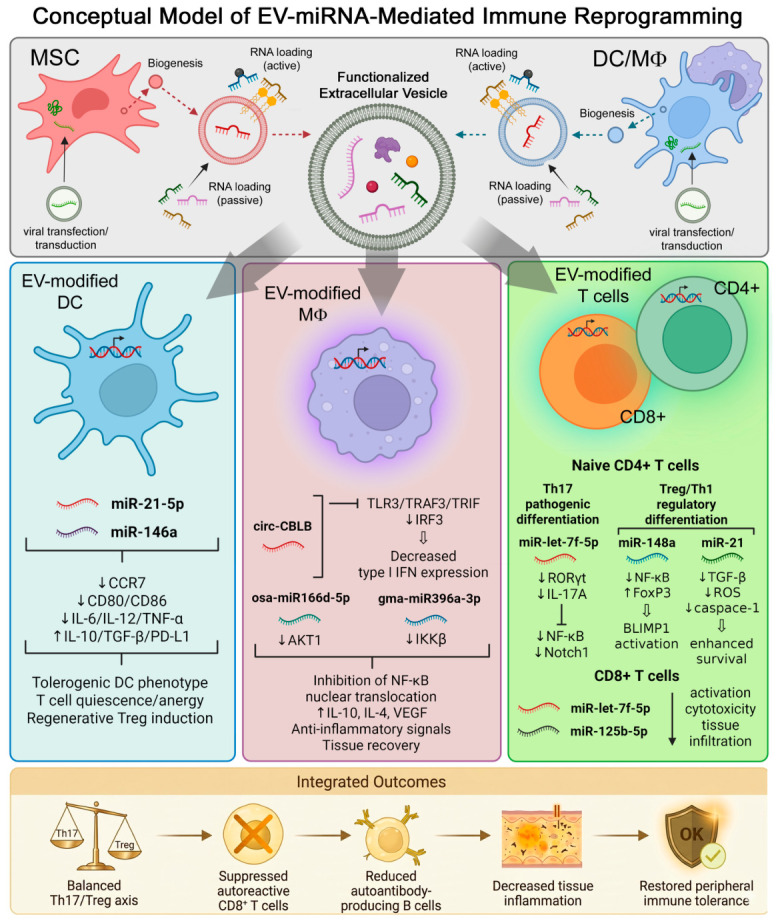
The conceptual model of RNA-loaded EV-based therapy for immune reprogramming in autoimmune diseases. The concept includes EV biogenesis, RNA loading (often facilitated by various methods like using magnetic beads or conjugation of RNAs with lipidated complexes), EV journey to the target cells, and RNA delivery mechanisms in immune modulation. The therapeutic journey of EVs begins with biogenesis from cells such as mesenchymal stem cell (MSC) or dendritic cell or macrophage (DC and MΦ, respectively), followed by their systemic distribution to reach various immune cell populations. In DCs, EV-encapsulated miR-21-5p and miR-146a induce a tolerogenic phenotype by downregulating CCR7 and CD80/CD86, leading to T cell anergy and Treg induction. In MΦs, functionalizing miRNAs include circ-CBLB, osa-miR-166d-5p, and gma-miR-396a-3p, which target various mechanisms like TLR3/TRAF3/TRIF, AKT1, and IKKβ. As a result, macrophages are polarized from M1 to M2 state, the production of type I IFN is decreased, nuclear translocation of NF-κB is inhibited, production of anti-inflammatory cytokines (IL-10, IL-4, VEGF) is elevated—altogether this promotes tissue healing and regeneration. In Naïve CD4+ T cells, let-7f-5p inhibits pathogenic Th17 differentiation, while miR-148a and miR-21 promote regulatory Treg/Tr1 differentiation by modulating NF-κB and TGFB1 signaling. In CD8+ T cells, miR-132-3p and miR-125b-5p reduce activation, cytotoxicity, and tissue infiltration. The integrated outcome of these mechanisms includes a balanced Th17/Treg axis, suppressed autoreactive T cells, and reduced autoantibody production, ultimately restoring peripheral immune tolerance and decreasing tissue inflammation. The dotted arrows coming from MSC or DC/MΦ (top part of image) highlight the “evolution” of EVs from biogenesis through engineering to the finally functionalized vesicle. The shadowed arrows show that the functionalized EV can modify activity of different target immune cells. The image was created in Biorender by Oleg Lookin (2026), https://app.biorender.com/illustrations/canvas-beta/69da6163cf04dfdc383d287a (accessed on 12 April 2026).

Figure 4 illustrates the conceptual model of RNA-loaded EV-based therapy for immune reprogramming in autoimmune diseases. The miRNA-charged EVs (passively or actively loaded) can then interact with various immune cells, including antigen-presenting cells or T cells. The EVs often have a complex cocktail of miRNAs, thus allowing for triggering different intra- and intercellular regulatory cascades simultaneously. The balanced action of the cascades can favor extinguishing inflammatory behavior and initiating recovery and regenerative processes.

While Figure 4 illustrates basic molecular pathways constituting a mechanistic framework of how RNA-loaded EVs may regulate autoimmune processes, Table 3 provides a detailed review of key studies related to the actual mechanisms in various types of autoimmune diseases. Importantly, we enlisted both the source of EVs and exact miRNA cargo as well as the putative molecular targets and mechanisms, which are utilized according to the reported findings.

To refine our understanding of the mechanisms of RNA-loaded EVs in autoimmune diseases, we also compared reported findings from available studies that utilized different types of RNAs, including miRNAs, lncRNAs, siRNAs, etc. (Table 4). Similar to Table 3, along with an exact RNA we provide here both its molecular target(s) and plausible mechanism(s) that are triggered by the RNA.

## 4. Further Considerations and Perspectives

Recent research has demonstrated the therapeutic use of EVs for delivering RNA, revealing the mechanism of action and regulatory capacity of EVs in the context of autoimmune diseases, such as cytokine signaling, immune tolerance, and tissue remodeling. Naturally released EVs possess a wide range of RNA cargo, and some of them may represent non-beneficial content for a target cell. For enhanced therapeutic purposes, EVs can be manipulated to carry specific RNA molecules using various methods that have been previously discussed. Despite low immunogenicity, stability, and homing ability of EVs, optimization of their targeting is necessary to enhance efficacy in delivering RNA to target cells, especially in clinical applications. To ensure the successful use of RNA-loaded EVs, current challenges and potential risks, as well as alternative sources of the EVs and further applications, need to be addressed.

### 4.1. Current Challenges and Potential Risks

A major challenge in the therapeutic application of EVs is their inherent heterogeneity, so EVs exhibit significant differences in their RNA content, surface phenotype, and potency. Indeed, conventional methods currently used in their isolation and purification often result in a mixture of different types of EVs, leading to a wide range of RNA content and biological activity. Moreover, in addition to cells, the EVs can be derived from different biological fluids such as the placenta, serum, breast milk, and plasma. The diversity of EV sources results in substantial heterogeneity in composition, function, and cellular origin, which hinders large-scale production and RNA loading efficiency [330]. Recent orthogonal analysis has further highlighted these inconsistencies in cargo loading, suggesting that the diversity of EV sources necessitates more rigorous quality inspection and standardized manufacturing protocols for large-scale production [331]. These limitations can be addressed by controlled modification of both exosomes and their RNA cargo content, alongside the implementation of standardized, current Good Manufacturing Practice (cGMP)-compliant manufacturing protocols to ensure reproducibility, traceability, and quality across batches.

MSCs of various origins are the most common source for EV collection. Specifically, BMSCs and human UCMSCs have emerged as the preferred source for therapy of autoimmune diseases. In addition, because MSC-derived EVs are more controllable and reproducible compared to body-fluid-derived EVs, they remain the common source for EVs in RNA-based therapy. This is particularly evident in the development of off-the-shelf products, such as small EVs enriched with miRNA-124a, which is the most abundant miRNA in the central nervous system, thus representing an important therapeutic agent for autoimmune disorders related to hyperactivity of microglia [332]. Notably, this is also a key regulator of hyperplasia of fibroblast-like synoviocytes and therefore a major component in such autoimmune states as rheumatoid arthritis, multiple sclerosis, and lupus [191]. These engineered cell-free products retain the therapeutic power of stem cells, such as inhibiting the migration of rheumatoid arthritis-related fibroblast-like synoviocytes without the risks of unwanted differentiation associated with live cell injections.

While synthetic delivery systems like lipid nanoparticles (LNPs) with defined chemical nature and structure have set a benchmark for RNA delivery, transitioning EV-based drug delivery to the clinic requires addressing unique biological complexities and high sensitivity to their environment [333]. Despite originating from the same parental source, minor changes in cultivation conditions, including growth media, temperature, and passage numbers, result in variation of the final product [333]. Because of these inherent variabilities, intermediate and final assessment of the physical and chemical properties of EVs is essential. Accurate assessment of EV purity and product characterization remains challenging, necessitating specific production processes and rigorous characterization strategies. Main characterization parameters include exosome marker detection, particle size, and morphology and composition analysis [334].

Purity is commonly assessed through measurement of total particle number per total protein or amount of protein with a particular size. While nanoparticle tracking analysis (NTA) is commonly used for this purpose, this technique presents inconsistent measurements depending on the manufacturer and operation conditions. Moreover, taking into account that size measurements of EVs alone are insufficient to assess the purity, another method such as size exclusion high-performance liquid chromatography emerges as an alternative approach that provides improved reliability [334]. Within a cGMP framework, such analytical variability must be minimized through validated, standardized assays to ensure reproducibility and regulatory compliance.

Next, significant technical challenges exist, especially regarding the techniques of EV isolation and purification. Currently applied techniques are often incapable of yielding highly pure forms and desired production levels. The shift toward clinical-grade production therefore requires a transformation in isolation technology. While laboratory-scale isolation relies heavily on ultracentrifugation, this method is characterized by low yields, high time consumption, and the requirement for large input volumes and parameters, including rotor type (fixed-angle vs. swinging-bucket), centrifugation time, and gravitational speed. New physical methods based on deep and prolonged freezing prior to ultracentrifugation have been proposed to increase EV yield as high as twofold compared to the conventional ultracentrifugation [335]. However, the use of conventional methods such as ultracentrifugation still fails to provide consistent batch production, necessitating a new technological approach for clinical-grade manufacturing. For instance, tangential flow filtration (TFF) represents a more efficient alternative method with significant advantages in purity, reproducibility, enhanced batch-to-batch consistency, and higher yield for large-scale production [336]. Moreover, three-dimensional (3D) culture systems for better spatially distributed production of EVs are being implemented [337]. While 2D cultures often struggle with limited cell-supernatant availability, 3D systems have shown superior results for EV concentration and yield, though research continues into how these methods alter the therapeutic cargo content. In addition, to facilitate translation from lab-scale to clinical-scale EV production, new advanced platforms such as hollow fiber and membrane-based bioreactors have been offered, which retain a canonical phenotypic profile and ensure the feasibility of post-isolation EV engineering. Being recognized as a key platform for cGMP-compliant EV production, they enhance scalability, reduce contamination risks, and improve reproducibility of EV yield and composition [338].

The challenge with large-scale production of EVs for clinical use can only be met through the implementation of rigorous quality control parameters, isolation techniques, and adherence to newly developed regulations such as Minimal Information for Studies of Extracellular Vesicles (MISEV) and current Good Manufacturing Practice (cGMP) guidelines [339,340]. In this context, cGMP-compliant manufacturing ensures strict control over production processes, including cell sourcing, culture conditions, isolation procedures, and batch-to-batch consistency, thereby guaranteeing product identity, purity, sterility, safety, and potency required for clinical application.

Besides the manufacturing challenges, the mechanistic complexity and action of RNA-loaded EVs can also be considered a significant biological challenge. Unlike single-agent therapeutics, their therapeutic outcomes are achieved through various cellular processes such as restoration of Th17/Treg balance, modulation of macrophage polarization¸ regulation of autophagy, inflammation-mediated pathways, and others. While this multi-target capacity underlies the therapeutic robustness of EVs, it complicates mechanistic dissection and target validation, thus representing a potential source of risks if applied in certain immune pathologies. Therefore, a more comprehensive understanding of molecular mechanisms and modes of action is required.

Some RNAs presented in EVs serve as biological contributors to the progression of autoimmune disease. Engineering EVs to enrich specific RNA cargo, combined with surface modifications to enhance targeting and stability, may offer promising strategies to counteract the pathogenesis of autoimmune diseases while ensuring target specificity. For example, surface engineering of EVs has shown a positive effect on their loading capacity/efficiency that promotes better activity on target cells, as has been demonstrated in recent studies [256,257]. However, chemical modification of EVs aimed at enhancing their efficiency and biodistribution may affect the translational application of the RNA-loaded EVs. In contrast to naturally produced EVs, engineered EVs may have certain immunogenicity and diminished biocompatibility, and their systemic administration may induce mononuclear phagocyte system and/or T cell activation that intensifies EVs clearance and degrades their accumulation in target tissues [255]. This problem raises a need for critical consideration of safety aspects. Not only chemical modification of EV lipid membrane, but also the use of potent regulatory RNAs as a cargo may pose a risk for off-target immune modulation or immune reprogramming. Additionally, the fact that some EV-encapsulated RNAs actively contribute to the progression of autoimmune diseases also raises the possibility that the lack of effective control over the cargo may actually contribute to a worsening of the autoimmune disease, not to a relief. Comprehensive profiling of RNA cargo in EVs, coupled with functional potency assays and long-term safety studies, is critical to mitigate these risks.

Finally, the scalability of the extracellular vesicle product for clinical applications represents a serious challenge still under resolution. As the source for MSCs (the main type of parent cell for EVs) is largely limited, it cannot be considered a proper regular source for large-scale EV production. Even if being cultured in vivo for gathering EVs, the cell material has limited expansion capacity and therefore must be replenished from time to time. Moreover, if taken from a given patient or a donor, the stem cells possess a unique secretory profile and therefore RNA cargo in the secreted EVs. This additionally limits their use in many patients with various pathological states and immune statuses. The personalized approach where RNA content of EVs is tightly adapted to the specific patient may indeed be considered the best choice, but it prevents scalability of EV production. On the other hand, if the EVs are produced from a biological medium in strict conditions with fully regulated cargo composition (which can be adjusted by the actual need), this may provide a solution for producing enough amount of EVs. The next subsection addresses the relatively new approach based on using non-human/non-animal sources for obtaining large amounts of EVs for therapeutic use.

### 4.2. Alternative Sources of Extracellular Vesicles

As mentioned before, the use of EVs derived from mammalian cells shows therapeutic efficacy but faces challenges with scalability and associated cost. Plant-derived EVs offer alternatives, being isolated from a natural source which is present in large volumes, like tea flowers and leaves or lemon citrus. It may serve as a drug-delivery platform for RNA cargo delivery and therapy targeted against autoimmune diseases [232] and in regenerative medicine, like healing of cutaneous wounds and diabetic wounds [341,342]. Moreover, tea-derived EVs can function as a natural vehicle for miRNA delivery with the ability to internalize by macrophages and reprogram inflammatory responses. For instance, tea-derived EVs can transport osa-miR166d-5p and gma-miR396a-3p, which consequently suppress NF-κB signaling by targeting AKT1 and IKKβ, thereby promoting M2 macrophage polarization and alleviating colitis [328]. However, the therapeutic use of plant-derived EVs remains nascent, limited by challenges in extraction and purification, and a limited understanding of their biogenesis and trafficking [232]. For example, the lipid membrane content differs notably between plant-derived EVs and those derived from animal sources—the key difference is the abundance of glycerol lipids and phospholipids but the lack of cholesterol in the plant-derived EVs [343]. In turn, plant-specific components of the lipid membrane—e.g., phosphatidic acid and phosphatidylcholine—may activate cellular mechanisms via pathways that are distinct from those derived from animals. In addition, different profiles in lipid content underlie different abilities for crossing of biological barriers and EV fusion to target cells.

More closely related to mammalian physiology are EVs derived from the milk of mammals, including bovine, sheep, and porcine—the most common types of livestock [344]. There are numerous studies that demonstrated efficacy of milk-derived EVs in various pathological states including autoimmune disorders [232,345,346]. The milk-derived EVs can be administered orally, which represents a much easier and completely non-invasive way compared to the systemic administration; importantly, this way of administration has been shown to be effective in an animal model of cancer, while displaying both beneficial and non-beneficial outcomes [347]. Of note, milk-derived EVs contain many autoimmune disease-related miRNAs, including miR148a, miR-200a and miR-223 [348]. Moreover, some of the miRNAs are highly conserved between mammals. For example, the milk of humans and bovines shares the structure of miR148a, which is known to be implicated in systemic lupus erythematosus [349]. Similarly, miR-223 is abundantly present in bovine milk and is involved in the regulation of macrophage polarization, thus highlighting the interest of using this miRNA for treating rheumatoid arthritis and other aggressive autoimmune states [350]. This evidence underlies the potential applicability of the animal milk-derived EVs in clinical use for patients with autoimmune disorders.

However, recent studies indicate that the bioavailability of orally administered milk-derived EVs depends essentially on the method of their isolation and purification. For example, a novel method of cryogenic freezing treatment showed better milk-derived EV crossing through the gastro-intestinal barrier compared to the conventional ultracentrifugation, where most of the EVs resided in the intestine [335]. Another study compared several methods of milk-derived EV isolation and purification and found that, if pretreated by rennet (an enzyme complex produced in the stomach of ruminants), they represent superior quality, stability, and limited aggregation [351]. Therefore, despite virtually unlimited availability for large-scale EV production, milk products need to be prepared specifically to obtain as large amounts as possible with as high quality as possible of EV-product, and this issue is still under development.

While plant- and milk-derived EVs are relatively widely used not only for treating various disease states but also for balancing metabolism and nutrition processes and for regenerative purposes, there are limited studies that utilized an RNA-preloaded format, especially for in vivo or clinical use. This is in part due to their lower loading capacity compared to MSC-derived EVs [352]. Accordingly, the reported efficacy of milk-derived EVs strongly depends on experimental settings. For example, bovine milk was used to obtain EVs cargoed by siRNAs to successfully treat hepatocellular carcinoma, but in in vitro conditions—therefore, no issues with crossing biological barriers were met there [348]. Similarly, despite innovations in producing milk-derived EVs with much better loading capacity (e.g., via conjugation of siRNA with cholesterol or with stabilized calcium phosphate nanoparticles), the testing of the efficacy remains largely in vitro [352,353]. Finally, despite the fact that milk- and plant-derived EVs may carry numerous molecular agents having immunosuppressing properties, their xenogeneic nature imposes a significant challenge due to the risk of immune hyperactivation of the host to the pathogenic material. Of note, EVs obtained from edible foods may have little immunogenicity because of regular consumption of these types of food during the lifetime. For example, the EVs from green tea, oranges, cabbage, ginger, grapes, etc., are successfully used in preclinical studies showing prominent anti-inflammatory potential and antioxidant activity [354,355,356]. Further translational studies are necessary for accumulating a pool of data and constructing a mechanistic framework for effective and safe usage of non-human milk- and plant-derived EVs in humans.

### 4.3. Prerequisites for Successful Clinical Application of Extracellular Vesicle-Based Therapies of Autoimmune Diseases

The elucidation of molecular complexities of EVs including the diversity of RNA content is still representing a critical prerequisite for their successful and robust use in diagnostic and therapeutic purposes. The development of sequencing techniques greatly contributes to the progression in this direction.

For example, studies have demonstrated that basophils in systemic lupus erythematosus (SLE) get activated and release elevated levels of miR-24550, which selectively increases B-cell activation through the suppression of KLF5, thereby worsening the condition of SLE [357]. Ding et al. (2020) have demonstrated that EVs secreted by synovial fibroblasts under low oxygen conditions lead to an increase in the levels of miR-424 [358]. This increases the progression of rheumatoid arthritis by compromising the Treg and Th17 balance and leads to an inflammatory response through the targeting of FoxP3. This suggests that miR-424 could serve as a potential therapeutic target for the treatment of rheumatoid arthritis. Also, neutrophil-derived EVs enriched in miR-30d-5p aggravate SLE immunological dysregulation by driving Th17 and Tfh activation and an increase in B-cell populations and pro-inflammatory cytokines while decreasing Treg amounts. Inhibition of miR-30d-5p reversed these processes, emphasizing its importance in the pathogenic immune responses [359]. EV-delivered miR-132-3p, which is of M1 macrophage origin, exacerbates lung injury in SLE-related diffuse alveolar hemorrhage, and inhibiting this miRNA may represent a promising therapeutic strategy for treating this state [360]. Investigations have demonstrated that tRNA-derived fragments (tRF) species are commonly found in EVs [361]. For example, tRNA-derived small RNA, tRF-His-GTG-1, plays a critical role in the pathology of SLE, and this particular tsRNA is mediated by platelet-derived extracellular vesicles, leading to a pro-inflammatory signal that increases the formation of neutrophil extracellular traps and the production of IFN-α [362]. Therefore, the inhibition of the action of this tsRNA has the potential for a new therapeutic approach to mitigate SLE by reducing the active inflammation and the autoimmune response, but their synthesis and function as exRNAs are comparatively understudied [363].

It should be noted that there are various hurdles associated with exosomal RNA sequencing (exoRNA-Seq), which include limited quantities of RNAs obtained during the isolation process, an increased risk of contamination with other extracellular RNAs, the absence of universally optimized library preparation protocols, and gaps in the bioinformatics tools [364]. In an attempt to resolve these hurdles, there is an urgent need to improve techniques to enable accurate and sensitive sequencing of exosomal RNAs [365]. Recent advancements in next-generation sequencing (NGS) platforms have emerged as a solution that enables the effective screening of RNA signatures for monitoring autoimmune disease progression [366]. Using next-generation sequencing, Selmaj et al. (2017) defined the global RNA profile of serum EVs and identified four specific miRNAs that were significantly downregulated in relapsing-remitting multiple sclerosis patients compared to healthy controls [367]. The total small RNA sequencing is an effective tool to capture the full RNA landscape within EVs and exosomes, allowing a comprehensive outlook for finding diagnostic and therapeutic markers [368]. In particular, Kakan et al. (2020) have investigated serum exosomal RNAs in a mouse model of Sjögren’s syndrome by the small RNA deep sequencing technique, which allowed them to identify upregulated miRNAs involved in the pathogenesis of this disease [369]. Collectively, these advancements demonstrate that overcoming the technical limitations of exoRNA-Seq allows for the precise mapping of miRNA dysregulation across various autoimmune pathologies, and this will greatly drive the translation of these findings to clinical application.

From a translational perspective, RNA-loaded EV-based therapy remains at an early developmental stage. For future clinical development, it is essential to resolve current restrictions associated with EV-based drug design. To date, there is a scarcity of clinical trials conducted to elucidate the role of RNA cargo-loaded EVs therapy for autoimmune diseases, highlighting a clear demand for validation studies to support their clinical application. Furthermore, establishing standardized dosing remains a hurdle, as EV potency is derived from a complex mixture of RNA and proteins rather than a single active ingredient. The current status of EV-based clinical trials, mostly in Phase I/II, highlights the need for long-term safety data to monitor for potential immune reprogramming. Addressing these regulatory and pharmacological gaps is critical for EVs to move beyond the ‘bench’ and into routine clinical use. The development of miRNA-EV-based therapy against autoimmune diseases faces difficulties that arise from several factors, including collective mRNA action to target autophagy genes, a sensitive regulatory landscape to stress stimuli and genetic polymorphisms, necessitating precise “miRNA control maps” for clinical use [370].

In addition to bulk and small RNA sequencing methods, advanced techniques that use high resolution have allowed a better understanding of the cell-specific and spatially resolved processes involved in the regulation through EVs. One such advancement in terms of high-resolution single-cell RNA sequencing (scRNA-seq) now allows researchers to obtain direct information about how therapeutic agents elicit molecular responses in cells. Through scRNA-seq, distinct cellular populations and their associated transcriptional responses in various biological processes can be identified [371]. Notably, the application of single-cell RNA sequencing has helped to unravel the heterogeneity of immune cells and their subsets implicated in autoimmune diseases. For example, in rheumatoid arthritis, different populations of synovial macrophages linked with inflammation or the remission phase were identified, with unique transcriptomes in each population [372].

Furthermore, integration-based computational methods provide additional insights into the biological interpretation of single-cell data. For instance, Shao et al. constructed a computational platform named miRTalk that combines information from single-cell transcriptomics with EV-derived miRNA-target interactions to uncover dynamic pathways of cell–cell communication [373]. These techniques allow the localization of the effects mediated by EV-RNAs to specific populations of immune cells that are modulated selectively via specific inflammatory signaling pathways, such as NF-κB and NLRP3 inflammasome activation.

Another emerging approach is that of spatial transcriptomics, which offers additional information on the immune response organization in tissues affected by disease. Spatial transcriptomics has already proven itself highly valuable in fields like developmental biology and oncology [374]. The implications of this technique clearly indicate that immune responses and their effectiveness depend on the type of cell and also have a specific spatial domain. This factor becomes extremely important for developing RNA drugs from EVs because it emphasizes the significance of localized actions. This high level of refinement would definitely contribute towards improving RNA-based therapeutic treatments using EVs for autoimmune disorders.

Looking forward, the convergence of EV biology with systems immunology, multi-omics profiling, and computational modeling is likely to accelerate the rational design of EV-based RNA therapeutics. Advances in single-vesicle analysis, synthetic biology-driven EV engineering, and theranostic EV platforms may enable personalized and disease-specific interventions. Ultimately, the successful clinical translation of RNA-loaded EVs will depend on the integration of mechanistic insight, rigorous manufacturing, optimal biosafety control, and regulatory clarity, positioning EVs as a transformative platform for precision immunomodulation in autoimmune diseases.

## Figures and Tables

**Table 1:** Summary of RNA cargo loading strategies and purification protocols for extracellular vesicles.

EV Origin	miRNA Cargo	Loading Method	Purification Method	Ref.
**miRNA: Endogenous loading**				
*Viral Transfection/Transduction*				
BMSCs	miR-124a	Adenoviral Transfection	Polymer Precipitation (Total Exosome Isolation Reagent)	[191]
BMSCs	miR-146a	Lentiviral Transfection	Differential Ultracentrifugation	[192]
BMSCs	miR-223	Lentiviral Transfection	Differential Ultracentrifugation	[193]
ADSCs	miR-20a	Lentiviral Transfection	Polymer-Based Precipitation (ExoQuick-TC)	[194]
*Non-Viral Transfection (based on Lipofectamine/RiboFECT* ^TM^ *)*				
BMSCs	miR-150-5p	miR-150-5p Expression Plasmid	Polymer Precipitation (ExoQuick-TC)	[157]
BMSCs	miR-223-3p	miR-223-3p mimics (Lipofectamine 2000)	Differential Ultracentrifugation	[195]
BMSCs	miR-151-5p	Cy3-miR-151-5p or Ad-miR151	Differential Ultracentrifugation	[196]
BMSCs	miR-367-3p	miR-367-3p mimics (RiboFECT^TM^ CP)	Reagent-accelerated (Exo precipitation reagent) ultracentrifugation	[197]
BMSCs	miR-515-5p	Direct exosome transfection with miR-515-5p mimics (Lipofectamine 2000)	Modified differential ultracentrifugation (lower g-force for final ultracentrifugation, but use of centrifugal filter device)	[198]
BMSCs	miR-21-5p	miR-21 mimic (Lipofectamine-2000) or a lentiviral vector	Differential Ultracentrifugation	[158]
BMSCs	miR-320a	miR-320a mimics (Lipofectamine 2000)	Differential Centrifugation → Filtration → Ultracentrifugation	[160]
BMSCs	miR-223	miR-223 mimics (Lipofectamine 2000)	Total Exosome Isolation Kit (Precipitation)	[199]
UCBMSCs	miR-19b-3p	CD4+ T cells transfection by Interferin siRNA transfection reagent (Polyplus)	Differential Ultracentrifugation	[200]
Plasma	miR-126-3p	miR-126-3p mimics (Lipofectamine 2000)	Differential ultracentrifugation	[201]
human UCMSCs	miR-140-3p	miR-140-3p mimics (Lipofectamine 2000)	Polymer Precipitation (ExoQuick-TC)	[202]
*Natural Enrichment*				
BMSCs	miR-205-5p	Naturally enriched as a result of the chondrogenic differentiation process	Total Exosome Isolation Kit (Precipitation) followed by incubation at low temperature and ultracentrifugation	[203]
GMSCs	miR-148a-3p	Naturally enriched	Differential Ultracentrifugation	[204]
MSCs	miR-196b-5p	Naturally enriched	Ultracentrifugation	[205]
**miRNA: Exogenous loading**				
human UCMCSs	miR-451a	Chemical Transporter/Exo Transporter Incubation of isolated exosomes with cargo and a specialized transporter kit	Differential Ultracentrifugation for exosome isolation, ultrafiltration followed by washing steps for purification of loaded exosomes	[170]
LGMSCs	miR-let-7f-5p	Exo-Fect^TM^ Exosome Transfection Kit	Modified Differential Ultracentrifugation with a 30% Sucrose/D_2_O Cushion	[167]
Stem cells from human exfoliated deciduous teeth	miR-29a-3p	Exo-Fect^TM^ Exosome Transfection Kit	Differential Ultracentrifugation with 30% Sucrose Cushion	[206]
**Other RNAs (lncRNAs, siRNAs, etc.): Endogenous loading**				
human MSCs	lncRNA Klf3-AS1	Naturally enriched	Polymer Precipitation (Total Exosome Isolation Reagent)	[207]
BMSCs	lncRNA SNHG7	Plasmid Transfection (Lipofectamine 3000)	Polymer Precipitation (Total Exosome Isolation Reagent) combined with Ultrafiltration	[208]
human and rat BMSCs	circFBXW7	Transfection (Lipofectamine 2000)	Differential Ultracentrifugation, characterization confirmed by NTA and Western blotting for markers CD63, CD81, and TSG101	[209]
human SMSCs	circEDIL3	Adenoviral Transfection (Lipofectamine 2000)	Differential Ultracentrifugation	[210]
HEK293T-derived engineered EVs	IL-10 mRNA	Aptamer-Mediated Selection: cell transfection with DNA aptamer (Lipofectamine 2000)	Differential Ultracentrifugation with RNase Treatment	[150]
**Other RNAs (lncRNAs, siRNAs, etc.): Exogenous loading**				
M2 macrophages	siRIPK3	Exo-Fect^TM^ Exosome Transfection Kit	Differential Ultracentrifugation	[169]
Milk-derived exosomes	TNF-α siRNA	Sonication, with ~21% loading efficiency	Differential Ultracentrifugation	[107]
Milk-derived exosomes	TNF-α siRNA (siTNF), specifically modified with a hydrophobic cholesterol moiety (siTNF-chol)	Passive Incubation	Differential Ultracentrifugation	[106]

Notes: ADSCs, adipose-derived mesenchymal stem cells; BMSCs, bone marrow-derived mesenchymal stem cells; EV, extracellular vesicle; GMSCs, gingiva-derived mesenchymal stem cells; IL, interleukin; LGMSCs, labial gland-derived mesenchymal stem cells; lncRNA, long non-coding RNA; miR or miRNA, microRNA; siRNA, small interfering RNA; SMSCs, synovial mesenchymal stem cells; TNF-α, tumor necrosis factor alpha; UCMSCs, umbilical cord-derived mesenchymal stem cells; UCBMSCs, umbilical cord blood-derived stem cells.

**Table 2:** Key cell-specific components—effector and target cells, signaling pathways, and molecular players—of most common autoimmune disorders that can be targeted by RNA-loaded extracellular vesicles.

Category	Autoimmune Disorder	Effector Cells	Target Cells	Inflammatory Dysregulation Pathway	Ref.
Systemic	Systemic Lupus Erythematosus	B cells, T cells, pDCs, neutrophils	plasma cells, Th17 T cells, macrophages	TLR signaling, BAFF, deficient apoptosis clearance → ↑ Type I IFNs + auto-antibody production (dsDNA) → ↑ IFN-α, IL-6, IL-17 → inflammatory cascades	[258]
Systemic	Rheumatoid Arthritis	DCs, activated T cells, macrophages, B cells	plasma cells, Th1 cells, Th17 cells, fibroblast-like synoviocytes, chondrocytes, osteoclasts	genetic and environmental factors → ↑ TNF-α, INF-γ, IL-1, IL-17, MMPs, RANKL → FLS dysregulation → ↑ Th17:Treg ratio (collagen-specific response) → collagen degradation	[259]
Systemic	Sjögren’s Syndrome	CD4+/CD8+ T cells, B cells, plasma cells, Th17 cells	salivary epithelial cells	MHC II/TCR activation + ↑ RORC → ↑ Type I IFNs → disruption of Th17 balance → ↑ secretion of CD8+ proinflammatory cytokines/chemokines + autoantibody production (looped action) → ↑ salivary epithelial cell apoptosis	[260]
Systemic	Systemic Sclerosis	pDCs, B cells, Th2 cells, CD4+/CD8+ T cells	fibroblasts, endothelial cells	genetic and environmental factors → MCP-1 and activation of immune cells → ↑ IL-4, IL-6 → TGF-β → ↓ fibroblast apoptosis + ↑ ROS → ↑ endothelial cell apoptosis and ET-1 + ↑ tissue fibrosis	[261]
Organ-Specific	Multiple Sclerosis	CD8+ T cells, M1 macrophages, B cells, perivascular DCs, Th1 cells, Th17 cells	astrocytes, microglial cells, oligodendrocytes	soluble mediators of inflammation → ↑ GM-CSF + STAT3 → Th17 pathogenicity → IFN-γ, IL-17 → myelin degradation	[262]
Organ-Specific	Crohn’s Disease	APCs, Th0/Th1/Th2 cells, NK cells, macrophages, Th17 cells	intestinal epithelial cells, smooth muscle cells, goblet cells	↓ regulation of intestinal bacteria → ↑ NF-κB and NOD2 + proinflammatory cytokines (IL-12, IL-23, IL-21, IL-4) → INF-γ, TNF-α → inflammation hyperactivation	[263]
Organ-Specific	Ulcerative Colitis	APCs, neutrophils, macrophages, Th1/Th9 cells, Treg cells, NK cells	intestinal epithelial cells and mucus layer	↓ regulation of intestinal bacteria → ↑ IL-36 + proinflammatory cytokines and cytotoxic factors (IL-6, INF-γ, TNF-α, IL-12, IL-23, IL-13) → NF-κB/MyD88-mediated inflammation hyperactivation	[264]
Organ-Specific	Autoimmune Hepatitis	APCs, Th0/Th1/Th2/Th17 cells, Treg cells, macrophages, plasma cells	hepatocytes	genetic and environmental factors, molecular mimicry → ↑ IL-4, IL-6, IL-12, TGF-β → NLRP3 inflammasome → hepatocyte pyroptosis	[265]
Organ-Specific	Psoriasis	plasma cells, macrophages, CD8+ T cells, Th17 cells	keratinocytes	genetic and environmental factors → keratinocytic IL37 → ↑ IFN-α, TNF-α, IL-23, IL-12 → JAK/STAT signaling → ↓ Treg suppressive function → dysregulation of endothelial cells and fibroblasts	[266]
Organ-Specific	T1 Diabetes Mellitus	APCs, B cells, CD8+ cytotoxic T cells, CD4+ T cells, M1 macrophages	pancreatic β-cells	genetic and environmental factors → ↑ β-cell autoantigen presentation by APCs → ↑ IL-1β, IL-2 → NF-κB → β-cell apoptosis	[267]
Organ-Specific	Graves’ Disease	DCs, plasma cells, T cells, Treg cells	thyroid follicular cells	↑ TSHR + TSI as autoantibody → DC activation by IgGs → disruption of Th1/Th2 balance and Tregs dysfunction → ↓ IL-2, IL-10, IL-35, TGF-β → APCs overactivation (looped action) → hyperthyroidism	[268]
Organ-Specific	Myasthenia Gravis	plasma cells, CD4+ T cells, Treg cells, Th17 cells, complement, myoid cells (early onset only)	postsynaptic myocytes in neuromuscular junctions	genetic and environmental, fetal abnormalities in thymus gland development → ↑ IL-6, IL-17, Il-21, IL-22 and impaired AChR presentation to complement → ↓ AChR binding to neuromuscular junction → focal myocyte damage	[269]

Notes: AChR, acetylcholine receptor; APC, antigen-presenting cell; BAFF, B-cell activating factor; DC, dendritic cell; dsDNA, double-stranded DNA; ET-1, endothelin-1; FLS, fibroblast-like synoviocytes; IFN, interferon; IL, interleukin; JAK/TYK, Janus kinase/tyrosine kinase; MCP-1, monocyte chemoattractant protein-1; MHC, major histocompatibility complex; MMP, matrix metalloprotease; MyD88, myeloid differentiation primary response 88; NF-κB, nuclear factor kappa-B; NK, natural killer cells; NLRP3, NOD-, LRR- and Pyrin domain-containing protein 3; NOD2, nucleotide-binding oligomerization domain-containing protein 2; pDC, plasmacytoid dendritic cell; RANKL, receptor activator of nuclear factor kappa-Β ligand; RIP2, receptor-interacting protein kinase 2; ROS, reactive oxygen species; STAT, signal transducer and activator of transcription; TCR, T cell receptor; TGF-β, transforming growth factor beta; Th, T helper cells; TLR, toll-like receptor; TSHR, thyroid stimulating hormone receptor; TSI: thyroid stimulating immunoglobulin. The horizontally aligned arrow shows that preceding part is affecting the following part. Up- or down-directed arrow indicates an increase or a decrease in the corresponding molecular factor.

**Table 3:** Summary of the mechanistic action of EV-derived miRNA in autoimmune diseases.

EV Origin	miRNA Cargo	Recipient Cell/Tissue	Direct Molecular Target(s)	Mechanistic Action	Disease Context	Ref.
BMSCs	miR-124a	Rheumatoid arthritis (RA) fibroblast-like synoviocytes (RA-FLSs)	Not defined	Cell-cycle arrest in G0/G1 phase, ↑ pro-apoptotic proteins (ex., Bax, Bid, Caspase 3/9), ↓ anti-apoptotic proteins (ex., Bcl-2) → RA-FLS apoptosis	Rheumatoid Arthritis (in vitro): counteract synovial hyperplasia and joint destruction	[191]
MSCs	miR-196b-5p	Activated dermal fibroblasts	COL1A2, TGF-βR1	↓ COL1A2 expression and TGF-β signaling → amelioration of tissue fibrosis	Systemic Sclerosis (mouse) and other fibrotic skin diseases to prevent or reverse organ and skin hardening	[205]
human UCMSC	miR-451a	RA synovial fibroblasts (RA-SFs)	ATF2	↑ ATF2 transcripts degradation and negatively regulates ATF2 expression → reduced proliferation, migration, and invasion of SFs	Rheumatoid Arthritis (CIA rats): reduce synovial hyperplasia and alleviate joint inflammation and destruction in a collagen	[170]
human UCBMSCs	miR-19b-3p	CD4+ T cells	KLF13	↓ expression of KLF13 mRNA → inhibits Th17 differentiation and promotes Treg development; Restores the Th17/Treg balance	Systemic Lupus Erythematosus (SLE) (patient-derived cells): suppresses the overactive immune response and reduces tissue damage	[200]
BMSCs	miR-150-5p	RA-FLSs, human umbilical vein endothelial cells (HUVECs)	MMP14, VEGF	Promotes degradation or represses translation of targeted mRNAs → reduces pro-inflammatory cytokine-induced migration, invasion, and tube formation by targeting MMP14, VEGF	Rheumatoid Arthritis (CIA mouse): reduce joint destruction, alleviate synovial hyperplasia, and mitigate clinical symptoms like hind paw thickness	[157]
BMSCs	miR-21-5p	FLSs	TET1	Targets TET1, ↓ 5hmC levels on the KLF4 promoter and KLF4 transcription → inhibition of FLS proliferation; ↓ pro-inflammatory cytokines IL-1β, IL-6, TNF-α	Rheumatoid Arthritis (in vitro, CIA mouse): reduces joint swelling, bone destruction, and inflammatory infiltration	[158]
human BMSCs	miR-320a	RA-FLSs	CXCL9	↓ CXCL9 mRNA expression → inhibition of synoviocyte proliferation, migration, and invasion; ↓ IL-1β, IL-6, IL-8 secretion	Rheumatoid Arthritis (in vitro, CIA mouse): to inhibit synovial activation and joint destruction	[160]
BMSCs	miR-223	Macrophages	NLRP3	Targets NLRP3 mRNA to inhibit inflammasome activation; ↓ IL-1β, TNF-α, IL-18 secretion	Rheumatoid Arthritis (in vitro, CIA rat): reduces joint swelling, synovial inflammation, and bone destruction	[199]
BMSCs	miR-223	Macrophages (involved in the liver’s inflammatory response)	NLRP3	↓ NLRP3 and caspase-3 → block of inflammasome activation and caspase-1 cleavage; ↓ IL-1β and IL-18 and alleviates liver inflammation	Autoimmune Hepatitis: protects the liver from immune-mediated damage and prevents the progression of fibrosis	[193]
BMSCs	miR-223-3p	Hepatocytes, immune cells (macrophages/T cells)	STAT3	Directly downregulates STAT3/p-STAT3 → reducing of IL-6, IL-1β and TNF-α; Restores immune balance by decreasing Th17 cells and increasing Tregs	Autoimmune Hepatitis (mouse): reduces liver injury, lowers transaminase levels (ALT/AST), and mitigates inflammatory lesions	[195]
BMSCs	miR-146a	Immune cells in colonic tissue	TRAF6, IRAK1	↓ TRAF6 and IRAK1 → inhibition of NF-κB activation (↓ p-p65 and p-IκBα); ↓ pro-inflammatory cytokines TNF-α, IL-6, IL-1β	Inflammatory Bowel Disease (TNBS rat): attenuation of experimental colitis	[283]
BMSCs	miR-151-5p	Host BMSCs	IL4Rα	↓ IL4Rα and mTOR signaling → restores host MSC differentiation from a pro-osteopenic to bone-forming phenotype	Systemic Sclerosis (mouse): rescues osteopenia, skin fibrosis, and autoimmune phenotypes	[196]
BMSCs	miR-214	Dermal fibroblasts	IL-33, ST2 (IL-33 receptor)	Directly ↓ IL-33/ST2 signaling → reduces fibroblast proliferation, migration, α-SMA expression, and collagen production	Systemic Sclerosis: attenuation of skin fibrosis and regulation of inflammatory–fibrotic crosstalk	[287]
BMSCs	miR-367-3p	Microglia	EZH2	Silences EZH2 → upregulating SLC7A11 and GSH synthesis, and ↓ ferroptosis in microglia	Multiple Sclerosis (mouse): alleviate Experimental Autoimmune Encephalomyelitis	[197]
BMSCs	miR-515-5p	RA-FLSs	TLR4	Targets TLR4 mRNA to suppress the NLRP3/GSDMD pathway → prevents pyroptosis, preserves mitochondrial integrity, and reduces IL-1β and IL-18	Rheumatoid Arthritis (CIA rat): reduce synovial inflammation and joint destruction by preventing synoviocyte death and mitochondrial damage	[198]
BMSCs	miR-205-5p	RA-FLSs and joint tissues	MDM2	Binds the 3′-UTR of MDM2 to inhibit MAPK/NF-κB signaling → reduces IL-1β, IL-6, TNF-α, MMP-1, and MMP-13, and alleviating synovial inflammation and cartilage degradation	Rheumatoid Arthritis (CIA mouse): alleviate joint destruction and systemic inflammatory responses	[203]
human GMSCs	miR-148a-3p	RA-SFs CD4+ T cells	IKKβ	↓ IKKβ/NF-κB signaling → restores Th17/Treg balance and ↓ T cell activation and pro-inflammatory cytokines, limits RA-SFs invasion to protect cartilage and bone	Rheumatoid arthritis (CIA mouse) and other autoimmune disorders	[204]
human UCMSCs	miR-140-3p	RA-SFs and joint tissues	SGK1	Directly silences SGK1 → inhibition of proliferation and migration; ↓ TNF-α and IL-1β and promotes RA-SFs apoptosis	Rheumatoid Arthritis (CIA mouse): reduces joint fibrosis, alleviates joint injury, synovial hyperplasia, and chronic inflammation	[202]
human UCMSCs	miR-203a-3p.2	Macrophages (RAW264.7 cells, THP-1-derived macrophages, mouse peritoneal macrophages)	Caspase-11 (mouse)/Caspase-4 (human) and downstream phosphorylated NF-κB	↓ casp11/4 expression → inhibition of noncanonical inflammasome activation and ↓ of macrophage pyroptosis, LDH release, and proinflammatory cytokines (IL-1β, IL-6)	Inflammatory bowel disease; attenuation of DSS-induced colitis via immune modulation	[276]
LGMSCs	miR-let-7f-5p	CD4+ T cells	RORC	↓ RORC mRNA → inhibition of differentiation of naive T cells into pathogenic Th17 cells and reducing the secretion of IL-17A; Restores the Th17/Treg balance	Sjögren’s Syndrome: alleviate salivary gland inflammation and xerostomia (dry mouth)	[167]
ADSCs	miR-20a	Renal podocytes and renal immune cells	Akt/mTOR	↓ Akt and mTOR phosphorylation → activation of autophagy (↑ Beclin-1 and LC3-II/I, ↓ p62); ↓ podocyte injury and decreases immune complex deposition	Lupus Nephritis (SLE condition): immunomodulation and renal tissue protection	[194]
Plasma	miR-126-3p	HUVECs	SLC7A5	↓ SLC7A5 expression → activation of the mTOR signaling pathway → inhibiting autophagy and oxidative stress; ↑ endothelial cell viability and angiogenesis	Systemic Sclerosis: therapeutic protection against vascular injury and microangiopathy	[201]
Tea-derived EVs	osa-miR166d-5p, gma-miR396a-3p	Macrophages	AKT1, IKKβ	Targets AKT1 and IKKβ → ↓ NF-κB signaling and promotes M2 macrophage polarization	Inflammatory Bowel Disease: attenuation of DSS-induced colitis	[328]
Stem cells from human exfoliated deciduous teeth	miR-29a-3p (endogenously enriched miRNA)	CD4^+^ T cells (Th1 cells)	T-bet	↓ T-bet expression → inhibition of Th1 differentiation and ↓ IFN-γ and TNF-α production	Sjögren’s Syndrome-induced hyposalivation via immunomodulation	[206]

Notes: APCs, antigen-presenting cells; ATF2, activating transcription factor 2; BMSCs, bone marrow-derived mesenchymal stem cells; CIA, collagen-induced arthritis; COL1A2, collagen type I alpha 2 chain; CXCL9, C–X–C motif chemokine ligand 9; DCs, dendritic cells; DSS, dextran sulfate sodium; EVs, extracellular vesicles; FLS, fibroblast-like synoviocytes; FoxP3, forkhead box P3; GMSCs, gingiva-derived mesenchymal stem cells; GSDMD, gasdermin D; HUVECs, human umbilical vein endothelial cells; IKKβ, inhibitor of nuclear factor kappa-B kinase subunit beta; IL, interleukin; IL4Rα, interleukin-4 receptor alpha; IRAK1, interleukin-1 receptor-associated kinase 1; KLF, Krüppel-like factor; LGMSCs, labial gland-derived mesenchymal stem cells; MAPK, mitogen-activated protein kinase; MDM2, mouse double minute 2 homolog; miR or miRNA, microRNA; MSCs, mesenchymal stem cells; mTOR, mechanistic target of rapamycin; NF-κB, nuclear factor kappa B; NLRP3, NOD-, LRR- and pyrin domain-containing protein 3; RA, rheumatoid arthritis; RA-FLS, rheumatoid arthritis fibroblast-like synoviocytes; ROS, reactive oxygen species; SF, synovial fibroblasts; SGK1, serum- and glucocorticoid-regulated kinase 1; SIRT1, sirtuin 1; SLE, systemic lupus erythematosus; SSc, Systemic Sclerosis; STAT3, signal transducer and activator of transcription 3; ST2, suppression of tumorigenicity 2; T-bet, T-box transcription factor TBX21; TET1, ten-eleven translocation methylcytosine dioxygenase 1; TGF-β, transforming growth factor beta; Th, T helper cell; TLR4, toll-like receptor 4; TNBS, trinitrobenzene sulfonic acid; TNF-α, tumor necrosis factor alpha; Treg, regulatory T cell; UC-MSCs, umbilical cord-derived mesenchymal stem cells; UCB-MSCs, umbilical cord blood-derived mesenchymal stem cells. The horizontally aligned arrow shows that preceding part is affecting the following part. Up- or down-directed arrow indicates an increase or a decrease in the corresponding molecular factor.

**Table 4:** Summary of the mechanistic action of EV-derived lncRNA/miRNA/mRNA/siRNA cargo in autoimmune diseases.

EV Origin	RNA Cargo	Recipient Cell/Tissue	Direct Molecular Target(s)	Mechanistic Action	Disease Context	Ref.
BMSCs	lncRNA SNHG7	HRMECs	miR-34a-5p → XBP1 → VEGF/TGF-β1	Sponges miR-34a-5p relieving repression of XBP1 → inhibition of EMT, angiogenesis, and tube formation under high-glucose conditions	Diabetic Retinopathy: therapeutic modulation of endothelial dysfunction and angiogenesis	[208]
BMSCs	lncRNA TUG1	CD4+ T cells	BLIMP1	Delivering of Lnc TUG1 from EV to CD4+ T cells → upregulates BLIMP1 → modulates Th17/Treg balance → decreases Th17 cells and increases Treg cells → alleviates RA symptoms	Potential therapeutic approach for treating rheumatoid arthritis	[321]
MSCs	tsRNA-21109	M1-type Macrophages	Rap1, Ras, Hippo, Wnt, MAPK, and TGF-β signaling pathways	↓ M1 markers (CD80, NOS2) and pro-inflammatory cytokines (TNF-α, IL-1β) → promotes M2 polarization	Therapeutic target for Systemic lupus erythematosus	[329]
BMSCs	circFBXW7	RA-FLSs	miR-216a-3p	Sponge miR-216a-3p preventing the miRNA from silencing HDAC4 → upregulation of HDAC4 suppresses the proliferation, migration, invasion, and inflammatory cytokine secretion of aggressive RA-FLSs	Rheumatoid Arthritis: reduce synovial hyperplasia and joint damage	[209]
human SMSCs	circEDIL3	RA-FLS and human dermal microvascular endothelial cells (HDMECs)	miR-485-3p → PIAS3 → STAT3 → VEGF axis	Transfer of circEDIL3 from EV to RA-FLS → circEDIL3 acts as a miRNA sponge for miR-485-3p → releasing PIAS3 inhibition → suppressing STAT3 activation and downstream VEGF expression → reduced angiogenesis	Anti-angiogenic and anti-inflammatory therapy for rheumatoid arthritis (in vitro and CIA mouse model)	[210]
RA-FLSs	circ-CBLB	M0-type macrophages M0 (M0 to M1 polarization)	TLR3/TRAF3/TBK1/IRF3 signaling axis	Directly binds TLR3 activating the TLR3-TRAF3 signaling cascade → TBK1 and IRF3 activation → enhanced M1 macrophage polarization (↑ CD80, CD86), and increased pro-inflammatory cytokine release (↑ TNF-α, IL-6)	Pathogenesis and immune amplification in rheumatoid arthritis; potential diagnostic biomarker and therapeutic target	[298]
HEK293T-derived engineered EVs	IL-10 mRNA	Colonic immune cells, predominantly macrophages (F4/80^+^)	IL-10 mRNA	EV-delivered IL-10 mRNA is translated in recipient cells → increases IL-10 levels and suppresses inflammatory responses in colonic tissue	Gene therapy approach for inflammatory bowel disease (DSS-induced colitis)	[210]
M2-type macrophages derived from RAW264.7 mouse macrophage cells stimulated with IL-4	siRIPK3	Hepatic Macrophages (liver-resident innate immune cells)	RIPK3	Targets and silences RIPK3 expression preventing necroptosis-related inflammation → reduces release of pro-inflammatory cytokines (e.g., TNF-α, IL-6) and chemokines → restores the Treg/Th17 balance	Immunotherapy for autoimmune hepatitis to provide a safer alternative to conventional steroid treatments	[169]
Milk-derived exosomes	TNF-α siRNA	human FLS-RA cells	TNF-α	Targets and inhibits TNF-α expression → reduces inflammation and cell proliferation	Rheumatoid Arthritis	[169]
Milk-derived exosomes	TNF-α siRNA	Intestinal Macrophages	TNF-α	Targets and silences TNF-α expression → reduces local inflammation and restores intestinal barrier integrity	Inflammatory bowel disease and colitis	[106]

Notes: BLIMP1, B lymphocyte-induced maturation protein 1; BMSCs, bone marrow-derived mesenchymal stem cells; CIA, collagen-induced arthritis; DSS, dextran sulfate sodium; EMT, endothelial-to-mesenchymal transition; EV, extracellular vesicle; FLS, fibroblast-like synoviocytes; HDAC4, class IIa histone deacetylase; HDMECs, human dermal microvascular endothelial cells; HEK293T, human embryonic kidney 293T cells; HRMECs, human retinal microvascular endothelial cells; IL, interleukin; IRF3, interferon regulatory factor 3; lncRNA, long non-coding RNA; MAPK, mitogen-activated protein kinase; miR or miRNA, microRNA; MMP, matrix metalloprotease; MSCs, mesenchymal stem cells; PIAS3, Protein Inhibitor of Activated STAT3; RA, rheumatoid arthritis; RA-FLS, rheumatoid arthritis fibroblast-like synoviocytes; Rap1, Ras-proximate-1/Ras-related protein 1; Ras, Rat Sarcoma; RIPK3, receptor-interacting serine/threonine-protein kinase 3; siRNA, small interfering RNA; STAT3, signal transducer and activator of transcription 3; TBK1, TANK-binding kinase 1; TGF-β, transforming growth factor beta; Th, T helper cell; TLR, toll-like receptor; TNF-α, tumor necrosis factor alpha; TRAF3, TNF receptor-associated factor 3; Treg, regulatory T cell; tsRNA, tRNA-derived small RNA; TUG1, taurine upregulated gene 1; VEGF, vascular endothelial growth factor; XBP1, X-box binding protein 1. The horizontally aligned arrow shows that preceding part is affecting the following part. Up- or down-directed arrow indicates an increase or a decrease in the corresponding molecular factor.

## Data Availability

No new data were created or analyzed in this study. Data sharing is not applicable to this article.

## Abbreviations

The following abbreviations are used in this manuscript:

AChR	Acetylcholine Receptor
ADSC	Adipose Tissue-Derived Stem Cell
AGO2	Argonaute-2 Protein
AHR	Aryl Hydrocarbon Receptor
ALT	Alanine Aminotransferase
APC	Antigen-Presenting Cell
Arc1	Activity-Regulated Cytoskeleton Associated Protein 1
AST	Aspartate Aminotransferase
ATF2	Activating Transcription Factor 2
ATG5	Autophagy-Related 5 Protein
BAFF	B-Cell Activating Factor
BLIMP1	B Lymphocyte-Induced Maturation Protein 1
BMSC	Bone Marrow-Derived Mesenchymal Stem Cell
CAR-T	Chimeric Antigen Receptor T Cell Therapy
CCR	C-C Motif Chemokine Receptor
CIA	Collagen-Induced Arthritis
circRNA	Circular RNA
COL1A2	Collagen Type I Alpha 2 Chain
CXCL9	C–X–C Motif Chemokine Ligand 9
DAMP	Damage-Associated Molecular Pattern
DC	Dendritic Cell
DPSC	Dental Pulp Stem Cell
dsDNA	Double-Stranded DNA
DSS	Dextran Sulfate Sodium
ECM	Extracellular Matrix
EDC	(1-Ethyl-3-(3-dimethylaminopropyl)carbodiimide)
EMT	Endothelial-to-Mesenchymal Transition
ERN1	Endoplasmic Reticulum To Nucleus Signaling 1
ESCRT	Endosomal Sorting Complex Required For Transport
ET-1	Endothelin 1
EV	Extracellular Vesicle
exoRNA-seq	Exosomal RNA Sequencing
EZH2	Enhancer of Zeste Homolog 2
FLS	Fibroblast-Like Synoviocyte
FoxP3	Forkhead Box Protein P3
GMP	Good Manufacturing Practice
GMSC	Gingival Mesenchymal Stem Cell
GSDMD	Gasdermin D
GSH	Glutathione
HDAC4	Class IIa Histone Deacetylase
HDMEC	Human Dermal Microvascular Endothelial Cell
HEK293T	Human Embryonic Kidney 293T Cell
HGF	Hepatocyte Growth Factor
hnRNPA2B1	Heterogeneous Nuclear Ribonucleoproteins A2/B1
hnRNPK	Heterogeneous Nuclear Ribonucleoprotein K
HRMEC	Human Retinal Microvascular Endothelial Cell
HUVEC	Human Umbilical Vein Endothelial Cell
ICAM-1	Intercellular Adhesion Molecule 1
ICOS	Inducible Costimulator Protein
IFNγ	Interferon γ
IKKβ	Inhibitor of Nuclear Factor Kappa-B Kinase Subunit Beta
IL	Interleukin
IL4Rα	Interleukin-4 Receptor Alpha
iNOS	Inducible Nitric Oxide Synthase
IP3R	Inositol 1,4,5-Trisphosphate Receptor
IRAK1	Interleukin-1 Receptor-Associated Kinase 1
IRF3	Interferon Regulatory Factor 3
JAK/TYK	Janus Kinase/Tyrosine Kinase
Jarid2	Jumonji AT-Rich Interactive Domain 2
KLF	Krüppel-Like Factor
LC3	Microtubule-associated protein 1A/1B-light chain 3
LGMSC	Labial Gland-Derived Mesenchymal Stem Cell
lncRNA	Long Non-Coding RNA
LNP	Lipid Nanoparticle
MALAT1	Metastasis Associated Lung Adenocarcinoma Transcript 1
MAPK	Mitogen-Activated Protein Kinase
MCP-1	Monocyte Chemoattractant Protein-1
MDM2	Mouse Double Minute 2 Homolog
MHC	Major Histocompatibility Complex
miR or miRNA	microRNA
MISEV	Minimal Information for Studies of Extracellular Vesicles
MMP	Matrix Metalloprotease
mRNA	Messenger RNA
MSC	Mesenchymal Stem Cell
mTOR	Mechanistic Target of Rapamycin
MVP	Major Vault Protein
MyD88	Myeloid Differentiation Primary Response 88
NF-κB	Nuclear Factor kappa B
NFAT5	Nuclear Factor of Activated T cells 5
NGS	Next-Generation Sequencing
NHS	N-Hydroxysuccinimide
NK	Natural Killer Cell
NLRP3	NOD-, LRR- and Pyrin Domain-Containing Protein 3
NOD2	Nucleotide-Binding Oligomerization Domain-Containing Protein 2
PAMP	Pathogen-Associated Molecular Pattern
PBMC	Peripheral Blood Mononuclear Cell
PD-L1	Programmed Death Protein Ligand 1
pDC	Plasmacytoid Dendritic Cell
PIAS3	Protein Inhibitor of Activated STAT3
RA	Rheumatoid Arthritis
RA-FLS	Rheumatoid Arthritis Fibroblast-Like Synoviocyte
RANKL	Receptor Activator of Nuclear Factor Kappa-Β Ligand
Rap1	Ras-Proximate-1/Ras-Related Protein 1
Ras	Rat Sarcoma
RIP2	Receptor-Interacting Protein Kinase 2
RIPK3	Receptor-Interacting Serine/Threonine-Protein Kinase 3
RORC	RAR-Related Orphan Receptor C
ROS	Reactive Oxygen Species
SAFB	Scaffold Attachment Factor B
scaRNAs	Small Cajal Body-Specific RNA
SF	Synovial Fibroblast
SGK1	Serum- and Glucocorticoid-Regulated Kinase 1
siRNA	Small Interfering RNA
SLC7A11	Solute Carrier Family 7 Member 11
SLE	Systemic Lupus Erythematosus
SNARE	Soluble N-Ethylmaleimide-Sensitive Factor Attachment Protein Receptor
SNHG16	Small Nucleolar RNA Host Gene 16
snRNA	Small Nuclear RNA
snoRNA	Small Nucleolar RNA
SMSC	Synovial Mesenchymal Stem Cell
ST2	Suppression of Tumorigenicity 2
STAT3	Signal Transducer and Activator Of Transcription 3
SYNCRIP	Synaptotagmin-Binding, Cytoplasmic RNA-Interacting Protein
T-bet	T-Box Transcription Factor TBX21
TBK1	TANK-Binding Kinase 1
TCR	T Cell Receptor
TET1	Ten-Eleven Translocation Methylcytosine Dioxygenase 1
TGF-β	Transforming Growth Factor Beta
Th	T Helper Cell
TLR	Toll-Like Receptor
TNBS	Trinitrobenzene Sulfonic Acid
TNF-α	Tumor Necrosis Factor Alpha
TRAF3	TNF Receptor-Associated Factor 3
Treg	Regulatory T Cell
tRF	tRNA-Derived Fragments
tsRNA	tRNA-Derived Small RNA
tRNA	Transfer RNA
TSHR	Thyroid Stimulating Hormone Receptor
TSHZ3	Teashirt Zinc Finger Homeobox 3
TSI	Thyroid Stimulating Immunoglobulin
TUG1	Taurine Upregulated Gene 1
UCMSC	Umbilical Cord Mesenchymal Stem Cell
UCBMSC	Umbilical Cord Blood-Derived Stem Cell
VAMP	Vesicle-Associated Membrane Protein
VEGF	Vascular Endothelial Growth Factor
WWC1	WW Domain-Containing Protein 1
XBP1	X-Box Binding Protein 1
YBX1	Y box Binding Protein 1
ZFP652	Zinc Finger Protein 652
